# Impacts of Nearby Algae on Recruitment Success and Early Microbiome Development of the Coral *Acropora cytherea*

**DOI:** 10.1111/1462-2920.70241

**Published:** 2026-01-15

**Authors:** Camille Vizon, Corentin Hochart, Pierre E. Galand, Maggy M. Nugues

**Affiliations:** ^1^ CRIOBE UAR 3278, EPHE‐UPVD‐CNRS‐PSL Perpignan France; ^2^ Environmental Biochemistry Group, Institute for Chemistry and Biology of the Marine Environment (ICBM), Carl von Ossietzky University of Oldenburg Oldenburg Germany; ^3^ Sorbonne Université, CNRS, Laboratoire D'ecogéochimie Des Environnements Benthiques (LECOB), Observatoire Océanologique de Banyuls Banyuls sur Mer France; ^4^ Laboratoire D'excellence « CORAIL» Moorea French Polynesia

**Keywords:** algae, bacterial communities, environmental effect, ontogeny, reservoirs, survival, Symbiodiniaceae

## Abstract

The persistence of coral reefs is dependent on the arrival and settlement of coral larvae followed by their post‐settlement growth and survival. Despite evidence showing that benthic algae have variable effects on corals, it is still unclear how benthic communities of the coral nursery habitat impact the early development of the coral microbiome and if these impacts relate to the survival and growth of newly settled corals. Here, we tested whether the survival and growth of *Acropora cytherea* recruits are impacted by surrounding algae, and whether specific algae influence their associated bacterial and Symbiodiniaceae communities. A 6‐month survey of coral larvae experimentally settled near different algae showed that crustose coralline algae enhanced recruit survival. However, despite variation in their microbiome, the presence of different algae did not impact the coral microbial community composition. The recruit microbiome was colonised by bacteria shared among all benthic substrates rather than bacteria unique to specific algae. Furthermore, the microbiome of coral larvae was different from that of the recruits. We conclude that the microbiome of corals in their early life stages is structured by host ontogeny rather than by their surrounding benthos, but that the surrounding benthos contributes to the transfer of opportunistic bacteria.

## Graphical abstract

Crustose coralline algae promote the survival and growth of coral recruits. However, these positive effects are not linked with the dynamics of the coral microbiome. Results reveal a transfer of opportunistic bacteria shared with all surrounding benthos, rather than a transfer of bacteria from specific algae to corals.

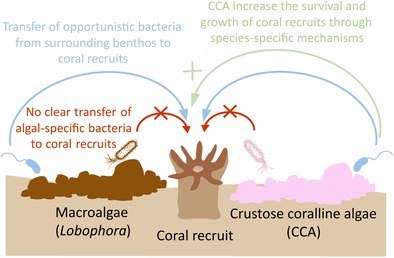

## 1 Introduction

Reef‐building corals live in symbiosis with a multitude of microorganisms, including dinoflagellates, viruses, fungi, archaea, and bacteria (van Oppen and Blackall 2019; Voolstra et al. 2024). These organisms are part of the coral holobiont and are intimately linked to the health of their host (Rohwer et al. 2002). Dinoflagellates in the family Symbiodiniaceae play a critical role in coral nutrition by providing most of the coral energy via photosynthate translocation (Muscatine and Porter 1977; Rosenberg et al. 2007). Coral‐associated bacteria are highly diverse (Galand et al. 2023) and also contribute substantially to host physiology and fitness by participating in nutrient acquisition, metabolic (re)cycling, and antimicrobial functions (Lesser et al. 2007; Raina et al. 2009, 2016). While bacterial communities are fundamental symbionts of corals, the process by which they are acquired by the coral host, particularly in larval and early juvenile stages, is still poorly understood. Corals can acquire their microbial symbionts horizontally from the environment, vertically from the parental colonies, or from a combination of both mechanisms (Bright and Bulgheresi 2010). The mode of Symbiodiniaceae transmission is closely associated with coral reproductive strategies. In broadcast spawners, Symbiodiniaceae are typically acquired from the environment, whereas in brooders, they can be transmitted from both parental colonies and the environment (Abrego et al. 2009; Quigley et al. 2017; Damjanovic, Blackall, et al. 2019). In contrast, the acquisition of bacteria is more variable. Bacterial communities of early coral life stages are more diverse than those of adult colonies, suggesting that the environment is an important reservoir of bacterial symbionts during development (Apprill et al. 2009; Littman et al. 2009; Lema et al. 2014; Epstein et al. 2019). Both seawater and sediment surrounding the coral host have been identified as potential reservoirs of microbes acquired throughout coral development (Patten et al. 2008; Apprill et al. 2009; Sharp et al. 2010; Bernasconi et al. 2019). However, benthic algae also harbour a large diversity of bacteria (Sneed et al. 2015; Jorissen et al. 2021; Hochart et al. 2024). Given their role as coral settlement substrata, they could provide a source of bacterial partners for newly settled corals via horizontal transfer, as well as influence the coral microbiome via a range of physical, chemical, or other microbially‐mediated mechanisms (McCook 2001; Barott and Rohwer 2012).

On coral reefs, intense competition occurs between sessile benthic organisms, particularly corals and algae that are two major components of the benthos (McCook 2001). Benthic algae are conventionally divided into functional groups, namely crustose coralline algae (CCA), macroalgae, and turf algae. CCA are commonly thought to have positive effects on corals and are generally associated with healthy reefs. These calcifying algae play a key role in the construction and maintenance of coral reefs (Steneck and Adey 1976). Their carbonate production can match or even exceed the contribution of corals to reef carbonate production, especially after major disturbances such as mass coral bleaching events (Cornwall et al. 2023). In addition, CCA contribute to reef resilience by providing settlement substrata for corals (Morse et al. 1988; Heyward and Negri 1999; Harrington et al. 2004; Jorissen et al. 2021), whilst preventing the colonisation of harmful macroalgae (Vermeij et al. 2011). Following major disturbances, the rapid growth of CCA on dead corals may enhance coral recruitment, leading to a faster recovery of damaged reefs (Perry and Morgan 2017). Inversely, macroalgae are often associated with compromised coral health and reef resilience. The cumulative impact of anthropogenic stressors (e.g., warming ocean temperatures, overfishing, pollution) has led to a decrease in the cover of reef‐building corals and calcifying algae and an increase in the cover of non‐calcareous macroalgae and algal turfs on coral reefs worldwide, with detrimental impacts on reef accretion (Hughes et al. 2007). Once established, macroalgae can reduce coral fecundity (Foster et al. 2008; Monteil et al. 2020) and recruitment (Kuffner et al. 2006; Box and Mumby 2007; Webster et al. 2015; Evensen, Doropoulos, Morrow, et al. 2019; Evensen, Doropoulos, Wong, and Mumby 2019) and cause further mortality or reduced growth in adult corals of certain species (Tanner 1995; Nugues and Bak 2006; Vega Thurber et al. 2012).

Despite evidence showing that benthic communities, such as CCA and macroalgae, have contrasting effects on corals, it is still unclear how benthic communities of the coral nursery habitat impact the early development of the coral microbiome and if these impacts relate to the survival and growth of newly settled corals. CCA microbial communities may play an important role in coral larval settlement (Negri et al. 2001; Sneed et al. 2015). Coral larvae have been shown to settle and metamorphose in response to specific bacteria and biofilms isolated from CCA (Negri et al. 2001; Tebben et al. 2011; Siboni et al. 2020; Petersen, Kellermann, et al. 2021). The inducing mechanism could be linked to secondary metabolites produced by the bacteria. Notably, several inducing molecules have been extracted from bacteria isolated from CCA (Petersen, Moeller, et al. 2021; Sneed et al. 2014, 2024). In addition, CCA host distinct microbial assemblages, with some species harboring bacteria promoting the survival and growth of coral recruits, while others host pathogens associated with coral disease (Sneed et al. 2015; Siboni et al. 2020). Similarly, indirect effects of macroalgae mediated through microbes are increasingly considered as an important mechanism by which macroalgae may affect corals (Smith et al. 2006; Barott and Rohwer 2012). Macroalgal assemblages alter the microbial communities of coral larvae and adults (Vega Thurber et al. 2012; Pratte et al. 2018; Clements et al. 2020; Pozas‐Schacre et al. 2024; Altman‐Kurosaki et al. 2025), induce coral disease and bleaching (Nugues et al. 2004; Vieira et al. 2016; Heitzman et al. 2023), and enrich overlying waters with potential pathogens and virulence genes (Barott et al. 2012; Kelly et al. 2014; Haas et al. 2016; Pozas‐Schacre et al. 2025). Several pathogens associated with coral diseases have been found on algal surfaces (Nugues et al. 2004; Sweet et al. 2013; Casey et al. 2014). Coral larvae may use differences in benthic bacterial community composition to assess the suitability of settlement substrates, selectively settling on algal species that harbor beneficial bacteria (Sneed et al. 2015). Furthermore, since the environment surrounding coral recruits can influence the composition of their microbiome (Hochart et al. 2024), the bacterial communities associated with settlement substrata could affect the health of coral recruits and serve as reservoirs for the development of their microbiome (Sneed et al. 2015). However, these hypotheses remain largely untested.

The objective of this study was to investigate the effects of nearby algae on the survival, growth, and microbiome dynamics of coral recruits. Larvae of the coral *Acropora cytherea* were settled ex situ on PVC rings inside which were embedded several patches from either one of four substrates: the two CCA species *Neogoniolithon* cf. *megalocystum* and *Lithophyllum* sp., the brown macroalgae *Lobophora* spp., or dead CCA (control). Substrates with newly settled corals were then deployed in situ for 6 months in the back reef of Moorea, French Polynesia. These algae were chosen for their ability to live and grow in semi‐cryptic to cryptic environments since coral larvae prefer to settle on cryptic surfaces. The two CCA species are known to induce coral settlement (Jorissen et al. 2020, 2021). *Lobophora* is an algal genus commonly found in French Polynesia (Vieira et al. 2022) and known to produce a variety of allelochemicals that can disrupt the coral microbiome (Birrell et al. 2008; Morrow et al. 2017; Fong et al. 2019). Given the potential positive role of CCA and the inhibitory capacity of *Lobophora*, we hypothesized that (1) recruit survival and growth would increase near the CCA species and decrease near *Lobophora*. Furthermore, we expected that (2) the early development of the coral microbiome would differ depending on the surrounding algae, and (3) that the bacterial communities associated with the surrounding algae could act as reservoirs for the coral recruit microbiome.

## 2 Materials and Methods

### 2.1 Experimental Overview

**FIGURE 1:**
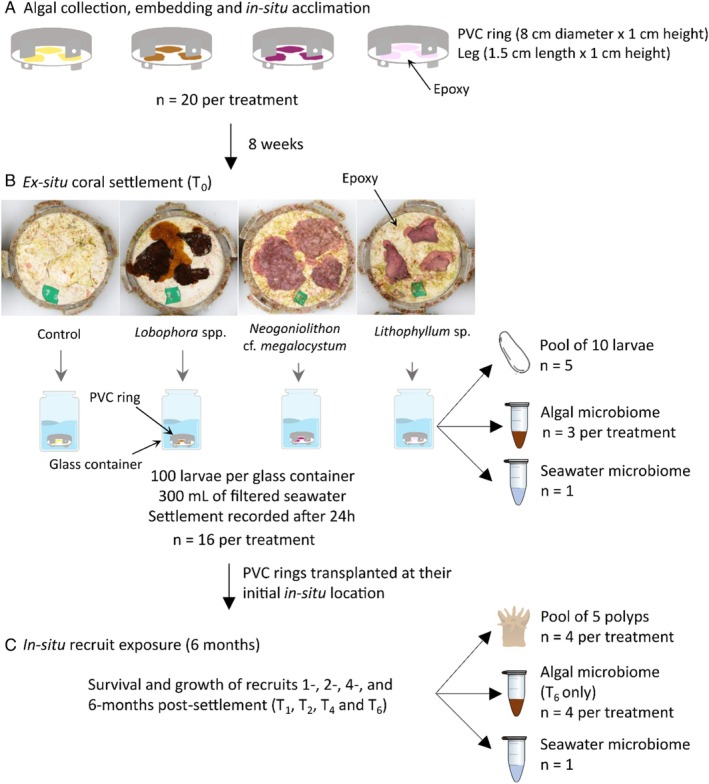
Experimental design and microbiome sampling. (a) Algal collection, embedding and acclimation. (b) Coral settlement experiment. (c) Post‐settlement experiment. PVC rings were oriented facing downward and held 1 cm above the reef substratum by four legs to create a subcryptic microhabitat (Figure S1).

The experiment involved 4 algal treatments: (1) a control composed of dead bleached CCA, (2) the macroalgae *Lobophora* spp., (3) the CCA *Neogoniolithon* cf. *megalocystum*, and (4) the CCA *Lithophyllum* sp., and consisted of three phases (Figure 1). First, the different algae were collected, embedded in epoxy inside PVC rings, and acclimated in the field for 8 weeks (Figure 1a). Second, the PVC rings were exposed to coral larvae *ex‐situ* to initiate settlement (Figure 1b). Settlement of larvae during this phase was assessed after 24 h. Third, the PVC rings were replaced in situ to follow the survivorship and growth of newly settled corals for a period of 6 months (Figure 1c). The microbiomes of corals, algae and seawater were sampled at different times throughout the experimental duration.

### 2.2 Algal Collection and Acclimation

Eight weeks before estimated coral spawning time, patches (ca. 5–6 cm^2^) of each algal species were collected using hammer and chisel at a ca. 1–2 m depth in the back reef of Moorea, French Polynesia (17°28′52.7″ S, 149°50′55.62″ W). *Lobophora* was followed by the suffix “spp.” due to the high diversity of species in the genus and the difficulty to distinguish between species in the field in French Polynesia (Vieira et al. 2022). The two CCA species were identified in the field according to Vizon et al. (2024) and Figure 3 therein. The suffix “cf.” was added to the *Neogoniolithon* species because there is no type sequence for *N. megalocystum* (Vizon et al. 2024). Patches were stored in separate zip lock bags filled with surrounding seawater and transported to the CRIOBE research station inside dark coolers. Three patches of the same algal species were embedded in epoxy (Minute Mend) within each PVC ring (80 mm diameter × 10 mm height) and left to harden for 24 h in flow‐through aquaria before being deployed in the field. The control treatment consisted of *N*. cf. *megalocystum* patches bleached for 48 h and soaked in freshwater for 12 h before embedding.

Embedded patches were left to acclimate for 8 weeks at the same site and depth they were collected from. To create a subcryptic microhabitat, PVC rings were secured at an upward angle between 10° and 30° from the horizontal, with algal patches facing the reef substratum (Figure S1). Four legs previously glued around the ring maintained a ca. 10 mm gap between the reef substratum and the algal surface, allowing for water exchange underneath the ring. Two opposite legs had a slot for attachment to the reef substratum with cable ties. To secure each ring, two holes were drilled into dead coral substratum using a pneumatic drill to insert cable tie mounts (Panduit product code HVMPM‐08‐C0), and each ring was secured using two plastic cable ties. Twenty replicates of each algal treatment were randomly spread over a ca. 200 m^2^ area and mapped for later retrieval.

### 2.3 Coral Collection and Larval Rearing

One to two days after the full moon in September 2021, mature colonies (*n* = 10) of *Acropora cytherea* were collected at ca. 2 m depth in two fringing reef sites of Moorea (17°33′13.4″ S 149°53′08.5″ W and 17°30′15.4″ S 149°45′51.7″ W) and transported to the laboratory. *A. cytherea* is a hermaphroditic broadcasting coral species, commonly found on the fringing reef of Moorea, that releases eggs and sperm in seawater a few days after the full moon of September/October in Moorea (Carroll et al. 2006). Colonies were placed into an outdoor flow‐through water table with constant aeration and maintained at ambient seawater temperature (ca. 27°C–28°C) using heaters. At 07:00 pm, colonies were checked and, when signs of imminent sperm‐egg bundle release were observed, they were isolated in 10 L buckets filled with seawater. For this study, we used larvae from one crossing that is, sperm of one colony mixed with eggs from another to avoid a host effect on the microbiome of the offspring. Sperm and eggs were isolated, mixed in one container with filtered seawater (GF/F, Whatman, 0.7 μm pore size), and left to fertilise for 2 h. Embryos were kept in 1.5 L closed containers without aeration on an orbital shaker at 30 rpm (Orbital shaker Stuart SSL1) under a 12:12 light: dark regime at a temperature range of 26°C–28°C for the first 3 days. Filtered seawater was changed every 12 h to remove dead larvae. Subsequently, larvae were moved into 6 L containers with aeration under the same light and temperature regimes with daily water changes. Larvae were used for the settlement experiment after 7 days at which time they were actively swimming in all directions and were assumed to have reached competency.

### 2.4 Settlement and Post‐Settlement Experiment

PVC rings were retrieved and brought back to the laboratory in individual zip lock bags inside coolers. Sixteen replicated rings of each treatment were photographed in high definition using a Canon EOS6D and a macro 100 mm lens, while submerged in seawater in individual containers. To ensure a consistent photographic setup, the camera and two halogen lamps were held on a dedicated copy stand. To initiate coral settlement, PVC rings were randomly placed in individual 700 mL glass containers filled with 300 mL of filtered seawater (GF/F, Whatman, 0.7 μm pore size), algal patches facing down (Figure 1b). A total of 100 competent larvae were added to each container. Containers were placed in a water bath held at 27°C under a 12:12 light:dark regime controlled with artificial lights. Settlement was assessed after 24 h using a dissecting microscope. Almost all (> 99%) larvae that had settled did so on the subcryptic side of the PVC rings where embedded algal patches were present. Settlement rates were expressed as the number of larvae that had settled and metamorphosed to the subcryptic side of the rings divided by the initial number of larvae added to the container and converted to percentage.

Coral recruits were mapped, their location relative to transplanted algae (on top or in contact vs. other) was recorded, and the PVC rings were returned to the field at their exact same position. Rings were brought back to the laboratory after 1, 2, 4 and 6 months (i.e., T_1_, T_2_, T_4_ and T_6_) to follow post‐settlement survival and growth through visual observations under a dissecting microscope. Survival was computed for each ring as the number of recruits remaining alive relative to the number of settlers at T_0_. Growth of each recruit was estimated by counting the number of polyps. Growth rate was computed as the (no of polyps at T_f_− no of polyps at T_i_)/(no of days between T_i_ and T_f_). Recruits that fused were counted as survivors, but they were not used in the growth data analysis (Putnam et al. 2020). PVC rings were used as replicates to avoid non‐independence of multiple recruits on a ring.

The percent cover of the algal patches was followed over time (T_0_ to T_6_) by tracing around the edge of the algal patches from the photographs using the ImageJ software. Percent cover of algal patches of each ring was calculated by dividing the total surface of algal patches by the subcryptic surface area of the ring. Additionally, the overall benthic community composition of the subcryptic surface area of each ring was quantified from the photographs at T_0_ and T_6_ by randomly placing 25 points and recording the dominant morpho‐functional group under each point (see Figure S2a for group names and definitions) using PhotoQuad (version 1.4) (Trygonis and Sini 2012).

### 2.5 Microbiome Sampling

The microbiome of corals was sampled at the larval stage at the beginning of the experiment (T_0_) and at the recruit stage 1‐, 2‐, 4‐, and 6‐month post‐settlement (Figure 1b,c). Due to small larval and recruit sizes, samples were obtained by pooling several larvae or recruits to have enough DNA for sequencing. At T_0_, 5 replicates each containing a pool of 10 larvae were collected. At T_1_, T_2_, T_4_, and T_6_, 4 replicates were collected for each algal treatment. Each replicate consisted of a pool of at least 5 polyps taken from 2 to 5 recruits chosen haphazardly across all rings from the same treatments. Due to this sampling procedure, not all rings were sampled, while some rings were sampled multiple times. Sampling at the recruit stage was conducted in the laboratory under a dissecting microscope after retrieval of PVC rings from the field. Coral recruits were carefully detached from the substratum with a sterile scalpel. Larval and recruit samples were rinsed three times in 0.2‐μm filtered autoclaved seawater to remove loosely associated microbial cells and subsequently stored into 2 mL cryotubes with 1 mL of DNA/RNA Shield (Zymo Research).

The algal microbiome was sampled at T_0_ (3 replicates) and T_6_ (4 replicates) by scrubbing using a swab, and then scraping the entire sub‐cryptic surface of the PVC ring using a sterile scalpel, after rinsing the surface three times with 0.2‐μm filtered autoclaved seawater. The swab and scraping of each ring were transferred into a 2 mL cryotube with 1 mL of DNA/RNA Shield (Zymo Research). At T_0_, rings that were sampled for the algal microbiome were discarded. At T_6_, rings were sampled after removing all coral recruits with a sterile scalpel under a dissecting microscope. Finally, the microbiome of in situ seawater was sampled at each time point (T_0_ to T_6_). 5 L of seawater was filtered sequentially through a 2.7 μm filter (GF/D, Whatman) and a 0.2 μm filter (Millipore Express +, Sigma). The 0.2 μm filter was transferred into a 2 mL cryotube with 1 mL of DNA/RNA Shield (Zymo Research). Each sample was stored at room temperature for 24 h and stored at −20°C before DNA extraction.

### 2.6 DNA Extraction, PCR and Sequencing

DNA extractions were conducted using the ZymoBIOMICS Miniprep kit (Zymo Research, Torrance, CA, USA). Briefly, samples were transferred into BashingBead Lysis Tubes (0.1 and 0.5 mm), and a mechanical lysis of 3 cycles of 60 s at a speed of 6 m s^−1^ was performed with a FastPrep instrument (Fast prep 24‐5G, MP Biomedicals). Samples were then extracted according to the manufacturer's protocol. A 5 min incubation time was performed after adding ZymoBIOMICS DNA Wash buffer 1, and DNA was eluted with ZymoBIOMICS DNase/RNase Free Water incubated at 40°C to improve DNA extraction. DNA concentrations were measured by fluorometric quantification on a Qubit Fluorometer using the Qubit dsDNA HS Assay (Thermo Fisher Scientific, Whaltham, CA, USA). No extraction blanks were included in this study, so it should be noted that some low‐abundance ASVs may represent contaminants introduced during the step of DNA extraction and sequencing (Fierer et al. 2025).

DNA samples were shipped to Integrated Microbiome Resource at Dalhousie University (Halifax, NS, Canada), where all steps of library amplicons preparation and sequencing were carried out. The V3‐V4 region of the 16S rRNA gene was amplified and sequenced using the forward primer 341F (5′–CCTACGGGNGGCWGCAG–3′) and reverse primer 805R (5′–GACTACHVGGGTATCTAATCC–3′) (Klindworth et al. 2013). The Symbiodiniaceae nuclear DNA ribosomal transcribed spacer 2 (ITS2) region was amplified and sequenced using the forward SYM‐VAR‐5.8S2 (5′–GAATTGCAGAACTCCGTGAACC–3′) and reverse primer SYM‐VAR‐REV (5′–CGGGTTCWCTTGTYTGACTTCATGC–3′). Algal and coral samples were sequenced with a targeted depth of 100 k reads/sample, and water samples with a targeted depth of 50 k reads/sample. Paired‐end sequencing (300 bp read length) was performed at Dalhousie University (Integrated Microbiome Resource) according to the facility's protocol.

### 2.7 Sequence Analysis

The 16S rRNA gene amplicon sequences were processed using the DADA2 pipeline version 1.8 (Callahan et al. 2016). Forward and reverse primers were removed, and sequences were filtered as follows: trimLeft = c(17,21), truncLen = c(260,220), maxN = 0, maxEE = c(2,2) and truncQ = 2. Sample sequences were dereplicated, paired reads were merged, and chimeric sequences were removed using the *DADA2* package. Taxonomic assignment was performed against the Silva v138.1 database. Prior to data analysis, the amplicon sequence variant (ASV) abundance table was filtered by removing eukaryotic ASVs (chloroplast and mitochondria) and ASVs found in less than 4 samples. The Symbiodiniaceae ITS2 was analysed with the SymPortal pipeline (Hume et al. 2019). The presence and abundance of ITS2 profiles within each Symbiodiniaceae family was estimated by recognising the co‐occurrence of defining intragenomic ITS2 sequence variants (DIVs) within samples and those in ITS2 Symbiodiniaceae database hosted by SymPortal. Combinations of different DIVs were then used to predict ITS2 profiles and placed in subgenera, subclades and so forth (Hume et al. 2019; Smith et al. 2020).

### 2.8 Data Analysis

All plots were drawn using the *ggplot* package (Wickham 2009) and all analyses were performed on R 4.5.1 (R Core Team 2021). Algal patch cover data did not meet the assumptions of normal distribution (Shapiro–Wilk's test) and homogeneity of variance (Levene's test). Temporal changes in algal patch cover for each individual alga were analysed using a Kruskal‐Wallis test with time (T_0_ to T_6_) as fixed factor, followed by Dunn's post hoc tests adjusted with the Holm method as appropriate (dunn.test package). Differences in coral settlement rates were tested using a one‐way Analysis of Variance (ANOVA) with algal treatment as fixed factor (*rstatix* package). Recruit survival and growth data did not meet the assumptions of normal distribution and homogeneity of variance. They were analysed using a Kruskal‐Wallis test with algal treatment as fixed factor for each sampling time (T_1_, T_2_, T_4_ and T_6_), followed by Dunn's post hoc tests adjusted with the Holm method.

Bacterial community data were first analysed across sample types (i.e., seawater, algae, coral larvae and recruits). Thereafter, algal and coral recruit microbiomes, as well as coral Symbiodiniaceae communities, were analysed separately across algal treatments and time. Differences in alpha diversity (Legendre and Legendre 2012) between sample types were tested using the Kruskal‐Wallis test followed by Dunn's post hoc tests adjusted with the Benjamini and Hochberg method (Benjamini and Hochberg 1995). Differences in alpha diversity between algal treatments and time were analysed using 2‐way parametric ANOVA followed by Tukey's HSD pairwise comparisons as appropriate (*rstatix* package). For the analysis of microbial composition, a Principal Coordinate Analysis (PCoA) ordination plot of Hellinger‐transformed Bray–Curtis dissimilarity matrices was constructed using the *ordinate* function of the *phyloseq* package (McMurdie and Holmes 2013). Differences in microbial composition were tested using Permutational Multivariate Analysis of Variance (PERMANOVA, Anderson et al. 2006) with the *adonis2* function of the *vegan* package (Oksanen 2016). Pairwise comparisons between group levels were performed using the *pairwise.adonis* function of the *pairwiseAdonis* package and adjusted with the Benjamini and Hochberg method (Martinez Arbizu 2020).

As the analyses revealed a strong effect of time on the coral microbiome, we identified the ASVs that best explained the difference in coral microbiome across sampling times using the similarity percentage (SIMPER) method (Clarke 1993), via the *simper* function of the *vegan* package. Additionally, we identified indicator ASVs which characterised each time point of the development of coral microbiome using the *multipatt* function of the *IndicSpecies* package (Cáceres and Legendre 2009). To increase robustness, ASVs with an abundance of less than 1% were removed from the dataset.

To assess temporal changes in potential functional traits of coral bacterial communities, we used the program PICRUSt2 (Langille et al. 2013; Barbera et al. 2018; Douglas et al. 2020). Based on the ASVs sequences and abundance profile, PICRUSt2 extrapolates KEGG Orthologs (KO; Kanehisa et al. 2012), Enzyme Commission number (EC number; Matsuta et al. 2013), and MetaCyc abundance pathways (Caspi et al. 2007). The NMDS ordination of MetaCyc pathways was constructed using the function *metaMDS* of the *vegan* package. Differences in MetaCyc pathways between time were tested using PERMANOVA. We then identified indicator MetaCyc pathways for three homogenous time groups using the *multipatt* function of the *IndicSpecies* package.

To determine if nearby algae were a source of bacteria for coral recruits, an upset plot was constructed using the *UpSetR* package (Conway et al. 2017) to visualise ASVs unique to, or shared between recruits and nearby algae from all treatments, as well as coral larvae and seawater. Algal, seawater and coral recruit datasets from the different sampling times were pooled for this analysis.

## 3 Results

### 3.1 Cover of Algal Patches and Other Benthos

After 8 weeks of acclimation, embedded patches of *Lobophora* spp., *N*. cf. *megalocystum*, and *Lithophyllum* sp. covered 46.24% (±2.53 SEM), 43.68% (±1.49), and 16.01% (±0.85), respectively, of the subcryptic surface area of the PVC rings in their respective treatments (Figures 1b and S2b). During the post‐settlement phase (T_0_ to T_6_), the cover of *Lobophora* spp. remained unchanged, while the cover of *N*. cf. *megalocystum* and *Lithophyllum* sp. increased and declined, respectively, to reach 69.88% (±4.40) and 7.31% (±2.15) at T_6_ (Table S1; Figure S2b). Thin turf algae (i.e., filamentous algae < 5 mm height) dominated the control treatment and remaining space in the three algal treatments at both T_0_ and T_6_ (Figure S2a). At T_6_, thick turf algae were present (ca. 15%–20% cover) in all treatments, while other groups, such as bryozoans, sponges, and other CCA, remained below 5% cover, except for other CCA in the control treatment, which averaged 12% cover.

### 3.2 Coral Settlement and Post‐Settlement

**FIGURE 2:**
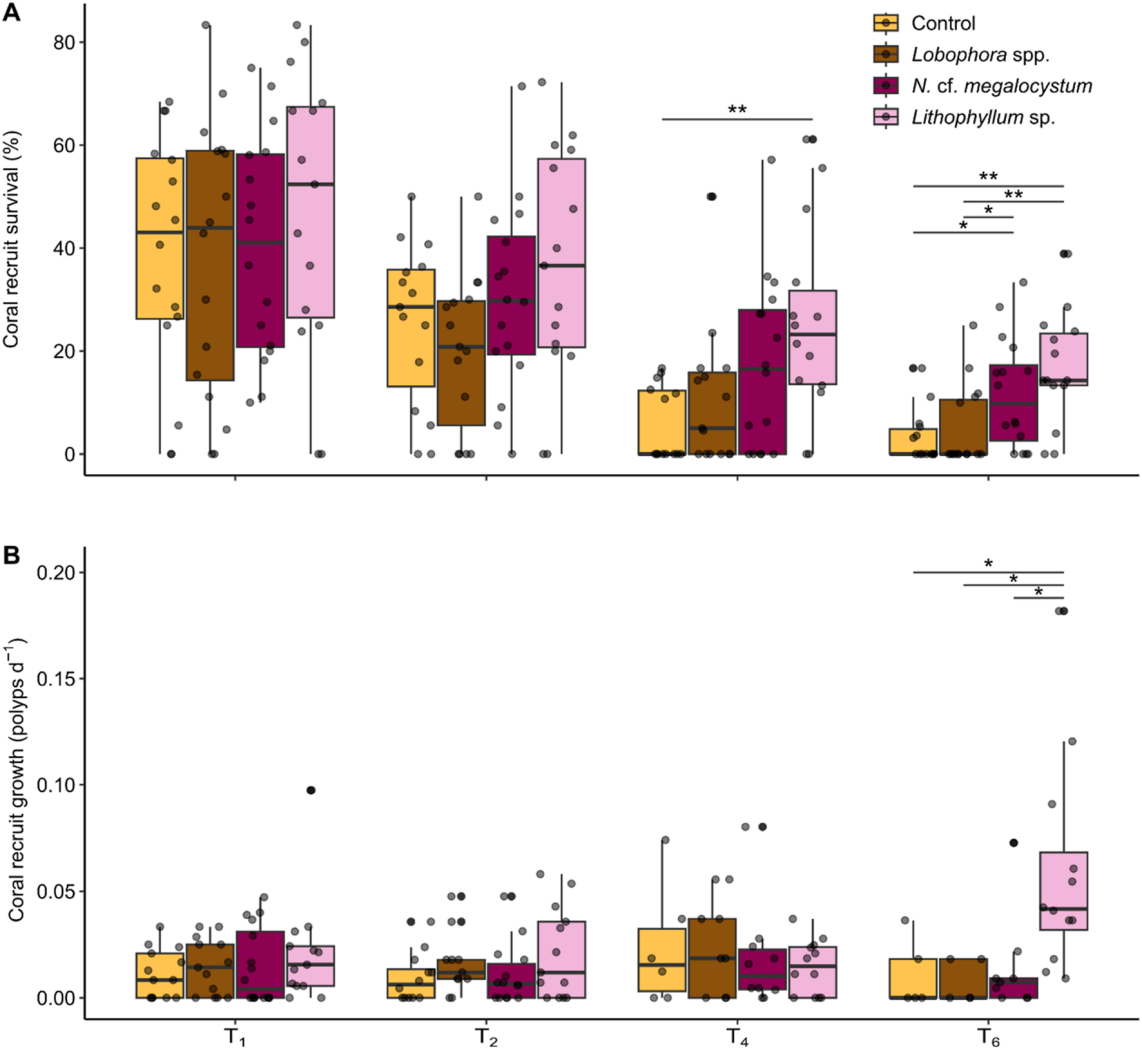
(a) Survival and (b) growth of *Acropora cytherea* recruits on the PVC rings as a function of time for the different algal treatments. The box plot horizontal bars show the median value, the box indicates the first and third QRs, and the whiskers indicate 1.5*IQR. Bars indicate significant differences between algal treatments for each sampling time according to Dunn's test adjusted with the Holm method (*p* < 0.05, **p < 0.01, ***p < 0.001; see Table S2 for exact values).

Coral settlement rates did not differ between algal treatments (one‐way ANOVA: *F*_3,59_ = 1.59, *p* = 0.201) and averaged 16.90% (±2.27) across treatments (Figure S3a). In contrast, recruit survival rates differed among algal treatments at T_4_ and T_6_ (Kruskal‐Wallis tests: *p* < 0.01; Table S2a). At T_4_, survival rates were 4.6 times higher in *Lithophyllum* sp. (23.75% ± 4.95%) than in the control (5.14% ± 1.75%) (Dunn's test: *p* = 0.009; Figure 2a). At T_6_, survival rates were 5.4 and 4.1 times higher in *Lithophyllum* sp. (15.44% ± 2.93%) and *N*. cf. *megalocystum* (11.73% ± 2.68%), respectively, than in the control (2.85% ± 1.22%) (Dunn's tests: *p* < 0.05). Survival rates were also higher in *Lithophyllum* sp. and *N*. cf. *megalocystum* than in *Lobophora* spp. (4.66% ± 2.68%). Recruit growth rates differed among algal treatments at T_6_ (Kruskal‐Wallis test: *F* = 13.76, *p* = 0.003; Table S2b). They were 5 times higher in *Lithophyllum* sp. (0.05 ± 0.01 polyps d^−1^) compared with all other treatments (0.01 ± 0.004 polyps d^−1^) (Dunn's test: *p* < 0.05; Figure 2b). Recruit survival and growth in *Lobophora* spp. did not differ from the control at any time point.

The high recruit survival rates in the *Lithophyllum* sp. treatment occurred despite the low cover of this CCA species on the subcryptic surface area of the PVC rings. Interestingly, 14% of the newly settled corals had settled on or in contact with *Lithophyllum* sp. in this treatment, while less than 1.4% of the corals were on or in contact with *N*. cf. *megalocystum* or *Lobophora* spp. (Figure S3a). Corals that settled on or in contact with *Lithophyllum* sp. had particularly high survival rates at all time points (e.g., 64% and 45% at T_4_ and T_6_; Figure S3b). When comparing recruit survival rates excluding corals that settled on or in contact with transplanted algae, differences in survival rates between *Lithophyllum* sp. and other treatments were no longer significant at both T_4_ and T_6_ (Table S2c), suggesting that the survival benefits in the *Lithophyllum* sp. treatment largely stemmed from the high survival rates of corals that settled on or in contact with *Lithophyllum* sp.

### 3.3 Microbiome Diversity Across Seawater, Algae and Corals

**FIGURE 3:**
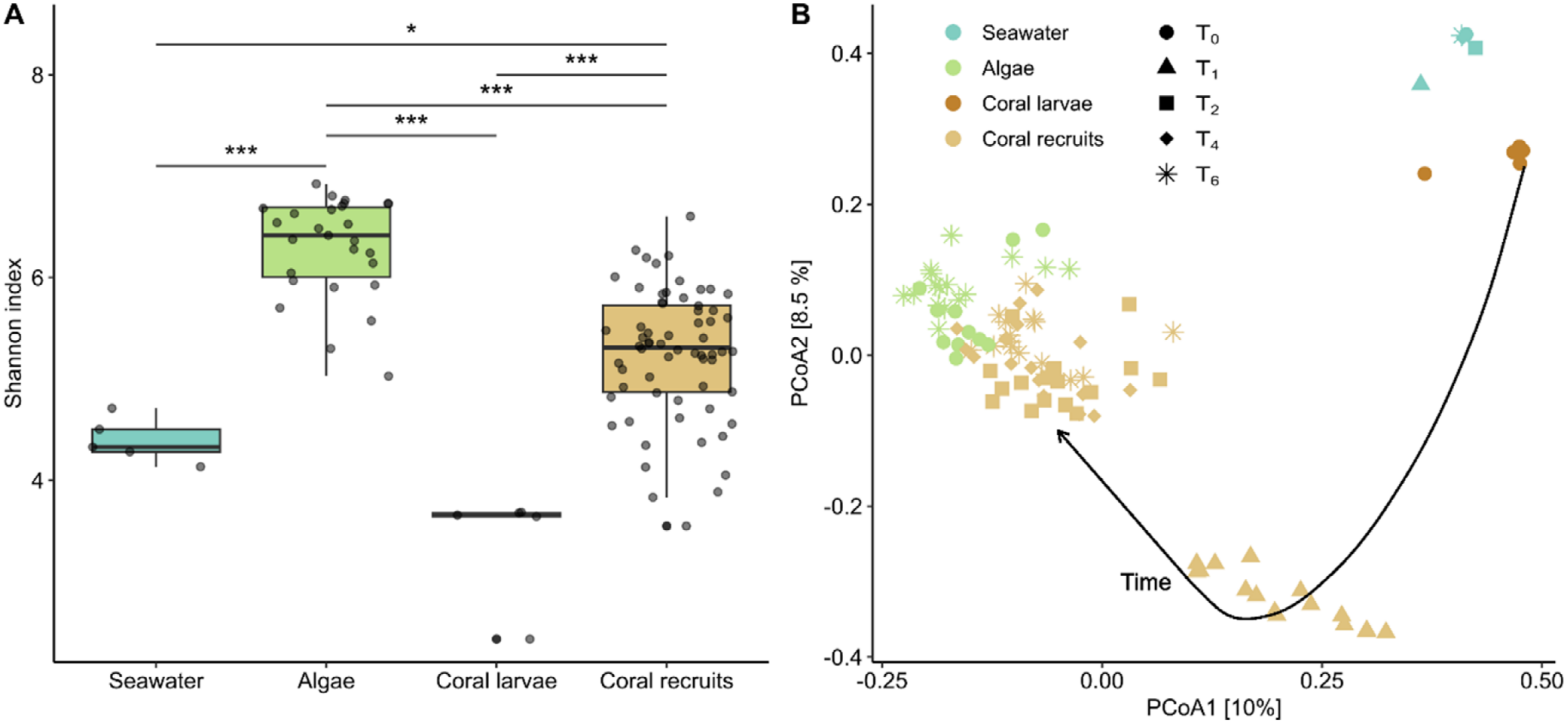
Microbial diversity and community composition of samples across seawater, algae and corals. (a) Alpha diversity (Shannon index). The box plot horizontal bars show the median value, the box indicates the first and third QRs, and the whiskers indicate 1.5*IQR. Bars indicate significant differences between sample types according to Dunn's post hoc tests adjusted with Benjamini and Hochberg (*p* < 0.05, **p < 0.01, ***p < 0.001; see Table S3 for exact values). (b) PCoA ordination based on Bray‐Curtis dissimilarities. Arrow indicates trajectory of the coral microbiome through time.

In total, we captured 28,532 ASVs from 102 samples across the four different sample types (6 seawater samples, 27 algal samples, 5 coral larval samples and 64 coral recruit samples). The number of reads per sample averaged 38,616. Rarefaction curves indicated that sequencing depth was sufficient to capture the majority of the microbial diversity in all samples, as we reached a plateau for each sample, except for one *Lobophora* sample at T_0_ which was excluded (Figure S4). Both microbial alpha diversity (Shannon index) and composition differed between sample types (alpha diversity: Kruskal‐Wallis test, *F* = 54.88, *p* < 0.001; composition: PERMANOVA, *F* = 8.61, *p* = 0.001; Table S3). Microbial communities of algae were the most diverse, followed by the ones of coral recruits, while coral larvae and seawater had the lowest microbial community diversity (Figure 3a). In the PCoA ordination plot (Figure 3b), microbial communities of coral larvae clustered near those of seawater, while microbial communities of coral recruits moved over time near those of benthic algae, supporting a distinct trajectory in the early development of the coral microbiome.

### 3.4 Algal Microbiome

**FIGURE 4:**
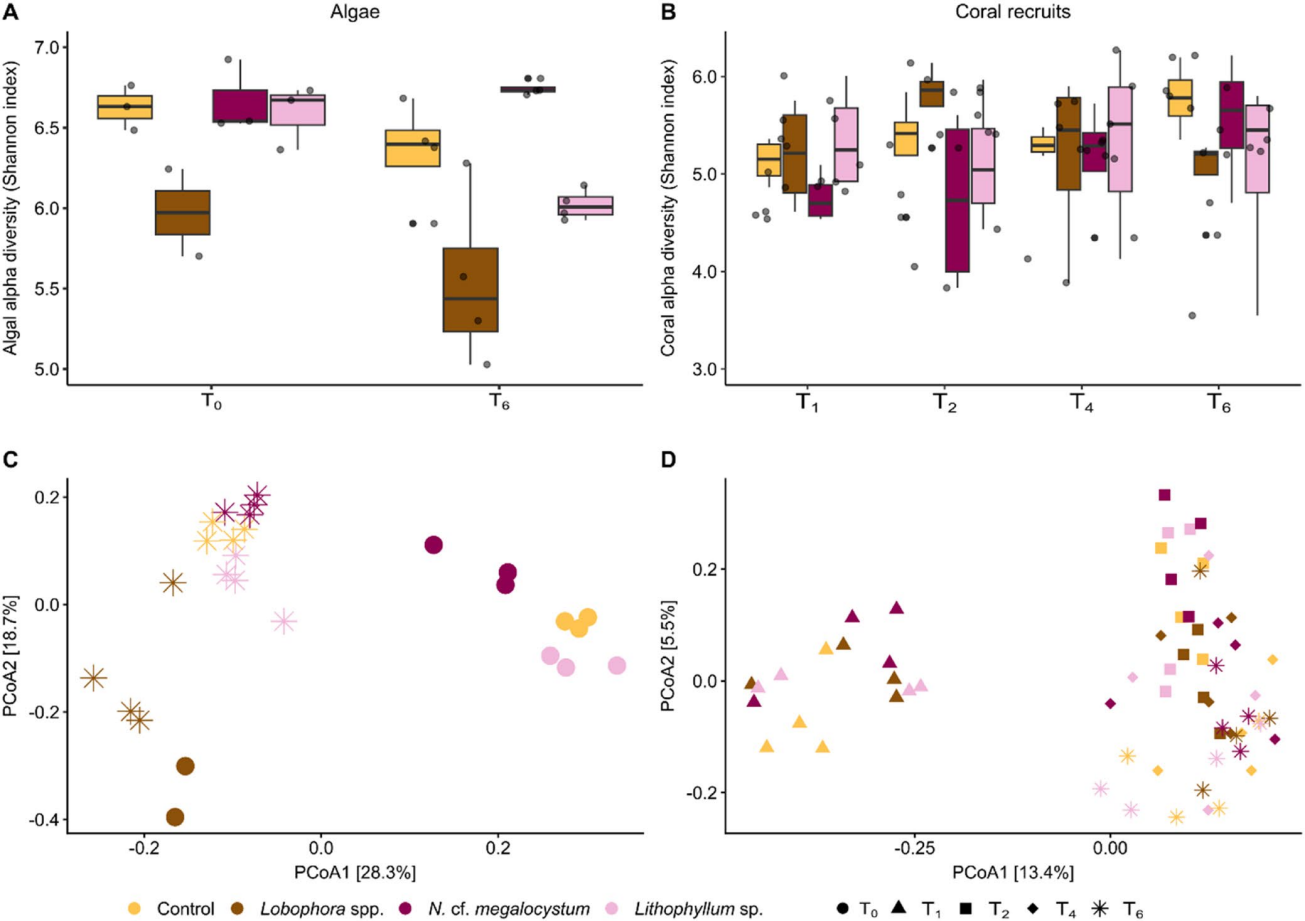
Microbial diversity and community composition of algal and coral recruit samples as a function of algal treatment and time. Alpha diversity (Shannon Index) of (a) algal samples and (c) coral recruit samples. The box plot horizontal bars show the median value, the box indicates the first and third QRs, and the whiskers indicate 1.5*IQR. PCoA ordination based on Bray‐Curtis dissimilarities of (b) algal samples and (d) coral recruit samples.

The alpha diversity of the algal microbiome varied between algal treatments and sampling time (2‐way ANOVA: treatment: *F*_3,19_ = 14.54, *p* < 0.001, time: *F*_1,19_ = 6.63, *p* = 0.019; Table S4a). Averaged across algal treatments, alpha diversity showed a 5.2% decline between T_0_ and T_6_ (Figure 4a). *Lobophora* spp. had a lower bacterial diversity than the two CCA species and the control (Tukey's HSD, *p* < 0.05). In addition, *N*. cf. *megalocystum* had a higher bacterial diversity than the *Lithophyllum* species. The composition of the algal microbiome differed between algal treatments and sampling time, with a significant interaction between both factors (PERMANOVA: treatment: *F* = 7.43, *p* = 0.001; time: *F* = 13.67, p = 0.001; treatment × time: *F* = 2.55, *p* = 0.001; Table S4b). In the PCoA ordination plot (Figure 4c), *Lobophora* spp. was clearly distinct from all other treatments, and *N*. cf. *megalocystum* differed from the control and *Lithophyllum* sp. (pairwise tests *p* < 0.05). All treatments, except *Lobophora* spp., differed between T_0_ and T_6_.

At the family level, ASVs affiliated to *Rhodobacteraceae* were found in abundance in all algal samples at both T_0_ and T_6_ (between 8% and 22%) and *Vibrionaceae* were found in all algal samples at T_6_ (average of 12%) (Figure S5). The control treatment was also dominated by *Nostocaceae*, *Sneathiellaceae* and *Stappiaceae* at T_0_ and by *Vibrionaceae* and *Kilonielaceae* at T_6_. In contrast, *Lobophora* spp. was associated with families belonging to *Pirellulaceae* and *Rhizobiaceae* at T_0_ and was most diverse at T_6_ with families affiliated with *Vibrionaceae* or *Nostocaceae*. At both time points, *N*. cf. *megalocystum* was associated with *Stappiaceae* and *Rhizobiaceae* (average of 8% and 13%, respectively), whereas *Nostocaceae* were abundant in *Lithophyllum* sp. (T_0_: 18% and T_6_: 14%). *Lithophyllum* sp. also had a high abundance of *Rhizobiaceae* at both time points and *Phormidiaceae* at T_6_.

### 3.5 Coral Microbiome

The alpha diversity of the coral recruit microbiome did not vary among algal treatments and sampling time, with no interaction between both factors (Table S5a; Figure 4b). In contrast, its composition differed among sampling times, with neither an effect of treatments nor an interaction between both factors (PERMANOVA: algal treatment: *F* = 1.15, *p* = 0.104; time: *F* = 4.62, *p* = 0.001; treatment × time: *F* = 1.03, *p* = 0.298; Table S5b). However, none of the pairwise comparisons for algal treatments were significant. Pairwise comparisons among sampling times showed all sampling months being different from each other. In the PCoA ordination plot (Figure 4d), T_1_ was clearly distinct from all other sampling times. In the subsequent analyses, the microbiome of coral larvae (i.e., the coral microbiome at T_0_) was added to the data set, and only the effect of time (i.e., T_0_ to T_6_) is discussed.

Coral larvae were associated with sequences affiliated with *Saccharospirillaceae*, *Sphingomonadaceae*, *Alteromonadaceae*, *Rhodobacteraceae*, and *Methylophagaceae*, all present at high (between 8% and 25%) relative abundance (Figure S6). For coral recruits, *Rhodobacteraceae* were found at high abundance across time (between 13% and 31%). *Alteromonadaceae* and *Nitrincolaceae* families were associated with 1‐month‐old recruits and decreased in abundance thereafter (T_1_: 29 to T_6_: 3% and T_1_: 15 to T_6_: 3%, respectively). *Kilonielaceae* were observed in 4‐ and 6‐month‐old recruits. Other ASVs belonging to *Simkaniaceae*, *Vibrionaceae*, *Rhizobiaceae*, or *Flavobacteriaceae* were observed at different time points.

**FIGURE 5:**
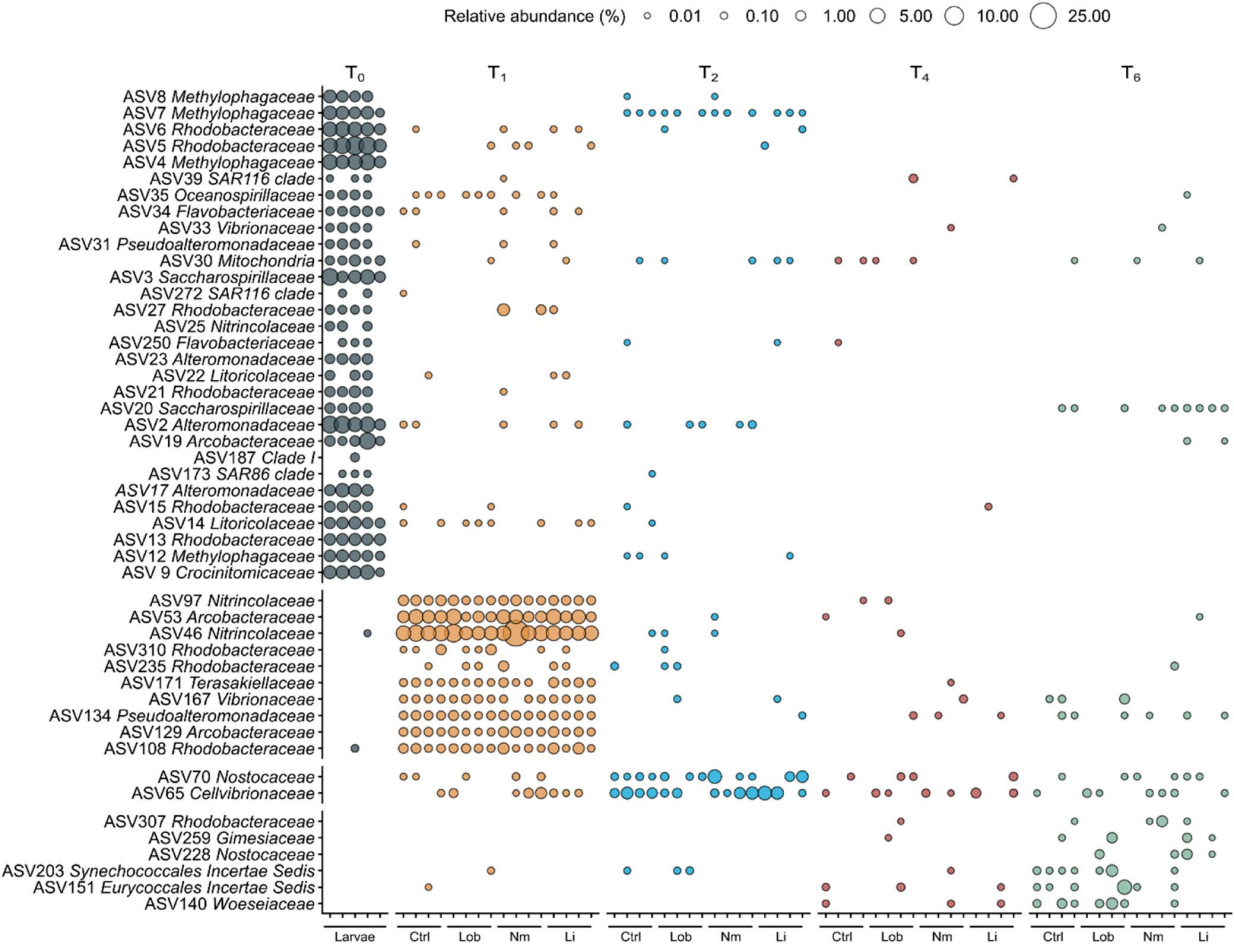
Indicator ASVs characterising coral larvae and coral recruits exposed to the different algal treatments as a function of time. Indicator ASVs are ordered vertically according to time. Ctrl, control condition; Li, *Lithophyllum* sp.; Lob, *Lobophora* spp.; Nm, *N*. cf. *megalocystum*.

SIMPER analyses indicated that *Rhodobacteraceae, Alteromonadaceae*, and *Methylophagaceae* contributed the most to the dissimilarity in bacterial communities between coral larvae and recruits (Supporting Information S1). *Alteromonadaceae*, *Nitrincolaceae*, and *Rhodobacteraceae* explained most of the dissimilarity between 1‐month‐old and 2‐ to 6‐month‐old recruits. The number of indicators ASVs varied between 30 for coral larvae and none for 4‐month‐old recruits (Supporting Information S2; Figure 5). For coral larvae, indicator ASVs were dominated by *Rhodobacteraceae, Alteromonadaceae, Methylophagaceae*, and *Saccharospirillaceae*. For 1‐month‐old recruits, the most significant indicator ASVs were assigned to *Nitrincolaceae* and *Arcobacteraceae*. Indicator ASVs for 2‐month‐old recruits were affiliated to *Nostocaceae* and *Cellvibrionaceae*, while the most significant indicator ASVs for 6‐month‐old recruits belonged to *Woeseiaceae* and *Synechococcales*.

### 3.6 Functional Potential of the Coral Microbiome

We found a total of 359 metabolic pathways from the 5 larval and 64 recruit samples. There was a significant effect of time on the composition of metabolic pathways of coral bacterial communities (PERMANOVA: time: *F* = 10.95, *p* = 0.001; Table S6; Figure S7). Pairwise comparisons revealed that metabolic pathways were different between coral larvae and other developmental stages (Pairwise tests, *p* < 0.05) and between 1‐month‐old recruits and other recruits (Pairwise tests, *p* < 0.001). The Indicator Species test revealed 19 indicator metabolic pathways (Figure S8; Supporting Information S3). Indicator metabolic pathways for coral larvae were associated with sucrose biosynthesis and phenylpropanoid compound degradation. For 1‐month‐old recruits, they were related to aromatic compound degradation and biosynthesis of secondary metabolites (terpenoids), while for 2‐ to 6‐month‐old recruits, they were associated with biosynthesis of chlorophyll A and vitamin B12, and sugar degradation. ASVs related to fatty acid biosynthesis were prominent for all recruit stages (Figure S8).

### 3.7 Shared and Unique ASVs Within and Between Coral and Algal Microbiome

**FIGURE 6:**
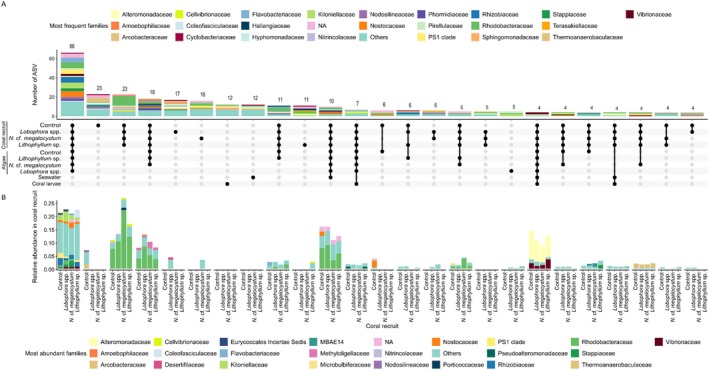
Upset plot showing (a) the number and (b) relative abundance (in coral recruits only) of ASVs unique to or shared between coral recruits and nearby algae from all algal treatments, as well as seawater and coral larvae. Sampling times were pooled. Only the first 25 combinations with the highest number of ASVs are shown.

The coral recruit microbiome harboured a high proportion of ASVs (66) shared by all four benthic substrates (Figure 6a). These ASVs represented between 18.9% and 20.3% of the sequences of each algal treatment (Figure 6b). They were mostly affiliated to Nostocaceae, Rhodobacteraceae, Rhizobiaceae, Kiloniellaceae, Flavobacteriaceae, PS1 clade and other families. 23 ASVs were shared among all recruits only, representing between 9% and 24% of their overall microbial community depending on algal treatment. Between 11 and 23 ASVs were unique to recruits from each treatment, but these represented only between 2.5% and 6% of the sequences.

A total of 6 ASVs were specifically shared between the control and its recruits, none were shared between *Lobophora* spp. and its recruits, 1 was shared between *N*. cf. *megalocystum* and its recruits, and only 2 were shared between *Lithophyllum* sp. and its recruits (Supporting Information S4). All of these ASVs represented less than 3% of the sequences of each algal treatment. ASVs shared between the control and its recruits were affiliated to the *Thermoanaerobaculaceae*, *Sneathiellaceae*, *Rhodobacteraceae*, and *Nostocaceae* families. Two ASVs were not identified due to their low similarity to database sequences. The ASV shared between *N*. cf. *megalocystum* and its recruits belonged to *Puniceicoccaceae*. ASVs shared by *Lithophyllum* sp. and its recruits were affiliated to *Cellvibrionaceae* and *Terasakiellaceae*.

### 3.8 Symbiodiniaceae Communities of Coral Recruits

Symbiodiniaceae were present in all coral recruits, but neither the diversity nor the composition of Symbiodiniaceae communities differed between algal treatments or time (Table S7; Figure S9a,b). Coral recruits were associated in majority with members of the *Durusdinium* clade, particularly the divisions D1, D4, and D6 (Figure S9c). *Cladocopium* (clade C) and clade A were also found.

## 4 Discussion

### 4.1 Crustose Coralline Algae Enhance Coral Recruit Survival

Although it is widely accepted that CCA can enhance coral larval settlement (Harrington et al. 2004; Ritson‐Williams et al. 2014; Jorissen et al. 2021), we failed to detect any differences in the settlement rates of *A. cytherea* larvae among algal treatments. Our PVC rings were not entirely covered by transplanted algae. Following the acclimation period of 8 weeks, the remaining space was occupied by an early successional stage community, mostly composed of sparsely distributed thin turf (Figures 1b and S3b). Such early successional stage community can offset settlement preferences of coral larvae for CCA (Elmer et al. 2018). Importantly, the presence of nearby CCA enhanced the survival of coral recruits at 4 and 6 months for *Lithophyllum* sp. and at 6 months for *Neogoniolithon* cf. *megalocystum*. We could not relate the survival benefits of the CCA with changes in the coral recruit microbiome, as discussed below. However, the survival benefits in the *Lithophyllum* sp. treatment could be explained by the high survival rates of corals (i.e., 64% and 45% after 4 and 6 months, respectively) that settled on or in contact with this species at relatively high abundance (i.e., 2.4%). Cryptic CCA, notably *L. prototypum* which is morphologically similar to *Lithophyllum* sp., are thought to favour coral settlement and post‐settlement survival, as they do not slough their tissue to get rid of epiphytes (Harrington et al. 2004; Price 2010). These species also form thin crusts and frequently lose space competition through overgrowth by thicker organisms (Arnold and Steneck 2011), which is consistent with the steady decline in *Lithophyllum* sp. cover in our study. Thus, corals that settled on or in contact with *Lithophyllum* sp. may have benefitted from reduced competitive overgrowth by the CCA.

Inversely, the cover of *N*. cf. *megalocystum* increased over time, and almost no larvae settled on or in contact with this species, suggesting alternative mechanisms. By occupying space, CCA can prevent the colonisation by turf algae and macroalgae that would otherwise harm corals (Vermeij et al. 2011). They can also promote healthier microbial and chemical microenvironments through distinct DOC release and thin diffusive boundary layer, including less labile DOC, less abundant and less active microorganisms and more dissolved oxygen (Haas et al. 2011, 2013; Jorissen et al. 2016; Wegley Kelly et al. 2022; Quinlan et al. 2025). However, we cannot exclude that, over a longer time period, coral recruit survival may decrease through competitive overgrowth by *N*. cf. *megalocystum*. Like the closely related species *N. fosliei* (Harrington et al. 2004), *N*. cf. *megalocystum* can slough its tissue (personal observations), and this thick‐crusted species can overgrow small‐sized recruits (Jorissen et al. 2020).

Contrary to expectations, coral recruit survival and growth did not decrease in the *Lobophora* treatment compared to the control treatment. Relatively few studies have explored the effect of *Lobophora* on coral recruit survival. Shading, but not contact, by *L. variegata* has been shown to reduce the survival of coral juveniles (Box and Mumby 2007). More recent work suggests that *Lobophora* has strong impacts on coral settlement, but relatively minor impacts on early post‐settlement survival, possibly because of low larval settlement in proximity to the alga (Morrow et al. 2017; Evensen, Doropoulos, Morrow, et al. 2019; Evensen, Doropoulos, Wong, and Mumby 2019). In our study, the cover of *Lobophora* remained unchanged throughout the study duration. The growth of the alga may have been limited by light due to the experimental cryptic conditions, dampening its negative effect on coral recruits through shading and direct contact.

### 4.2 Different Algae Do Not Impact the Coral Recruit Microbiome

We hypothesized that the early development of the coral microbiome would differ depending on the different algae. However, despite significant variations in the microbiome of nearby algae, the presence of different algae did not impact the coral microbial community diversity and composition. These results are consistent with studies investigating the development of bacterial communities across coral early life stages in different rearing conditions. For instance, recruits of the coral *Pocillopora acuta* harboured dynamic and diverse assemblages, which were not influenced by the presence of adult corals (Damjanovic, Blackall, et al. 2019). Likewise, the uptake and structure of bacterial communities in early life stages of the coral *Acropora digitifera* were consistent, regardless of exposure to seawater or sediment bacterial communities (Bernasconi et al. 2019).

During their first year of development, corals acquire a multitude of microorganisms and exhibit a highly diverse and heterogenous bacterial community (Littman et al. 2009; Zhou et al. 2017; Epstein et al. 2019; van Oppen and Blackall 2019). After 1 year, coral juveniles undergo a winnowing process where alpha diversity decreases and composition becomes stable until the adult microbiome establishes (van Oppen and Blackall 2019). We suggest that the highly diverse microbiome of coral recruits may have offset specific fluctuations in the algal microbiome and that different algae are more likely to impact a more mature coral microbiome. In another study, the microbial community of *Pocillopora* coral recruits under 25 mm in size varied depending on their surrounding substrate (i.e., CCA vs. well developed turf; Hochart et al. 2024). Since our 6‐month‐old recruits were less than 10 mm in size, the difference between this study and ours could be due to different recruit ages, as well as distinct coral species or reproductive strategies, or a combination of factors, including age and size. In the adult stage, most studies on coral‐algal interactions document significant changes in coral‐associated microbial diversity and composition in contact with different algae (Vega Thurber et al. 2012; Beatty et al. 2018; Clements et al. 2020; Fong et al. 2020; Briggs et al. 2021).

### 4.3 The Surrounding Benthos Contributes to the Transfer of Opportunistic Bacterial

In our study, very few ASVs were shared exclusively between the microbiome of a unique algal treatment and its coral recruits, providing no evidence of clear transfer of abundant bacteria from specific algae to corals that may contribute to the overall coral recruit microbiome. These results are consistent with a previous study comparing coral recruit microbial communities with those of surrounding algae (Hochart et al. 2024). In contrast, a large diversity of bacteria associated with coral recruits was shared across all four algal treatments and absent in coral larvae or seawater. These taxa represented ca. 20% of the sequences from each algal treatment. In fact, the coral microbiome followed a distinct trajectory of change from a microbial community close to that of seawater at the larval stage to one close to that of nearby algae within less than 6 months post‐settlement. Therefore, coral recruits may acquire some opportunistic and ubiquitous bacteria from the surrounding benthos during the first few months of their development. These bacteria may take advantage of a new ecological niche without being necessarily detrimental to coral health. However, the transitory nature and functional role of these bacteria in coral holobionts remain to be defined. Some of these bacteria, notably those belonging to the families *Rhodobacteraceae* and *Rhizobiaceae*, have been reported in coral early life stages (Bernasconi et al. 2019; Damjanovic, Menéndez, et al. 2019) and could contribute beneficially to biological processes, such as antimicrobial activity (Raina et al. 2016) and nitrogen fixation (Sharp et al. 2012; Lema et al. 2014).

### 4.4 Ontogenic Changes in the Coral Microbiome

Our results demonstrated that the microbiome of *A. cytherea* recruits was driven by host ontogeny rather than the experimental treatments. The microbiome of coral larvae was different from that of the recruits, which is similar to what has been observed in other coral species (Lema et al. 2014; Zhou et al. 2017; Bernasconi et al. 2019; Tignat‐Perrier et al. 2025). More specifically, significant changes in microbial composition and functional prediction were observed between coral larvae and one‐month‐old coral recruits, suggesting changes in metabolic requirement during growth (Bernasconi et al. 2019). The metamorphosis of coral larvae into a sessile polyp is an energetically costly process through the initiation of calcification and the biosynthesis of proteins needed for development (Edmunds et al. 2013; Huffmyer et al. 2025). Metamorphosis is coupled with ontogenetic alterations that lead to the formation of complex niches (i.e., coral skeleton, coral tissue, mucus) (Sharp et al. 2012; Lema et al. 2014; Zhou et al. 2017). This process is likely to favour colonisation by diverse and distinct bacterial communities (Apprill et al. 2016).

*Rhodobacteraceae*, *Alteromonadaceae* and *Methylophagaceae* contributed to the differences between coral larvae and recruits. The *Rhodobacteraceae* family is commonly found in the early life stages of several coral species (Apprill et al. 2009, 2012; Sharp et al. 2012; Damjanovic, Menéndez, et al. 2019; Bernasconi et al. 2019). Members of this family have been involved in the degradation of dimethylsulfoniopropionate (DMSP) (Raina et al. 2013) and the production of antimicrobial compounds against pathogens, and shown to play a protective role for coral larvae (Ceh et al. 2012; Sharp et al. 2012). Moreover, they may be involved in vitamin biosynthesis needed for coral growth (Li et al. 2022). Members of *Alteromonas* were present at a high abundance (ca. 17%). Members of this genus may provide nitrogen for the coral host and protection against pathogens (Ceh et al. 2013; Lema et al. 2014). Finally, the presence of *Methylophaga* sp. in coral larvae could be linked with dimethylsulfide (DMS) degradation (Raina et al. 2013). Recently, this strain has been involved in the synthesis of coral settlement cues (Siboni et al. 2020). Changes in the abundance of *Methylophaga* sp. have been related to the presence of Symbiodiniaceae which metabolise DMSP and other bacterial taxa that degrade DMS and DMSP, such as *Alteromonas* or *Pseudomonas* (Raina et al. 2010).

We observed that the inferred microbial pathways of coral larvae were enriched in sucrose biosynthesis and degradation. *A. cytherea* larvae are aposymbiotic (i.e., free of Symbiodiniaceae). Thus, they may require other symbionts for energy and heterotrophic feeding (Huffmyer et al. 2025). In our study, a decrease in the relative abundance of these pathways was observed after colonisation by Symbiodiniaceae, suggesting that photosymbionts can satisfy the energy requirements of the host. Further studies are needed to identify the bacterial strains associated with the increase of these pathways in coral larvae and to elucidate the nutritional exchanges between coral larvae and bacteria.

The families *Alteromonadaceae* and *Nitrincolaceae* were abundant in one‐month‐old coral recruits, along with inferred pathways associated with secondary metabolite biosynthesis and aromatic compound degradation. Members of *Nitrincolaceae* have been involved in the production of secondary metabolites (Ul Karim et al. 2022). Biosynthesis of secondary metabolites may inhibit the growth of coral pathogens (Ritchie 2006; Peixoto et al. 2021; Li et al. 2022). Additionally, the synthesis of secondary metabolites protects corals from environmental stress (Modolon et al. 2020). Finally, inferred pathways related to cobalamin (vitamin B12) and creatinine degradation were enriched in 2‐month‐old recruits. Vitamins are essential for the growth of coral host and Symbiodiniaceae, which are both auxotrophs for vitamins (Smith 2018). Cobalamin is an essential cofactor involved in the biosynthesis of amino acids (Peixoto et al. 2021) and methionine (Matthews et al. 2020). The acquisition of microbial symbionts may thus provide a source of vitamin for Symbiodiniaceae and coral host (Smith 2018). Creatinine degradation pathway may be associated with Symbiodiniaceae acquisition. In fact, this pathway provides nutrients, such as carbon and nitrogen, for Symbiodiniaceae (Matthews et al. 2020). It should be noted that these pathways were inferred from 16S sequences and need to be confirmed by metagenomics.

## 5 Conclusion

This study provides new insights into the role of benthic communities in the early development of the coral microbiome. Although nearby CCA promoted the survival of coral recruits, we could not link these positive effects with the dynamics of the coral microbiome. Similarly, we did not observe an influence of the different algae on the coral recruit microbiome, or a clear transfer of bacteria from specific algae, including CCA, to corals. Differences in bacterial community composition at the ASV level were driven by holobiont age rather than experimental conditions and reflected the evolving energetic and metabolic requirements of the recruits. However, we found that coral recruits harboured a large diversity of bacteria shared across all algal substrates, and not present in specific algae, coral larvae or seawater. These results support that the surrounding benthos may contribute to the horizontal transfer of opportunistic bacteria in the first year of coral development. Future investigations should focus on the functional roles of bacteria acquired during the early stages of coral development to determine if these communities contribute to recruit survival and growth.

## Author Contributions

M.M.N., P.E.G., and C.V. conceived the experiment. C.V. performed the experiment, collected the field data and carried out the DNA extraction. C.V., C.H., and P.E.G. analysed the data. C.V. and M.M.N. drafted the manuscript, and all authors contributed to revisions thereafter and gave final approval for submission.

## Funding

This work was supported by Agence Nationale de la Recherche, ANR‐18‐CE02‐0009‐01.

## Conflicts of Interest

The authors declare no conflicts of interest.

## Supporting information


**Supporting Information: S1.** Similarity analysis (SIMPER) between coral larvae and other time points.
**Supporting Information: S2.** Characteristic AVSs associated with coral larvae and recruits for the different sampling time.
**Supporting Information: S3.** Characteristic pathways associated with coral larvae and recruits for the different sampling time.
**Supporting Information: S4.** Details of shared ASVs between coral recruits and their algae. Shared ASVs between the control condition and their recruits are in yellow, between *N*. cf. *megalocystum* and their recruits in purple and between *Lithophyllum* sp. and their recruits in pink.

**Figure S1:** Field photograph of PVC ring (80 mm diameter × 10 mm height) with embedded patches. PVC rings with algae were facing the reef substratum.
**Figure S2:** Benthic community composition and algal patches cover on the inner surface of the PVC rings. (a) Benthic community composition at T_0_ and T_6_ for the different algal treatments. Thin and thick turf algae were defined as filamentous algae with heights of < 5 mm and ≥ 5 mm, respectively. Macroalgae were defined as non‐filamentous, anatomically complex algae. CCA = Crustose coralline algae. Values were assessed from 25 randomly chosen points on each ring using the PhotoQuad software. (b) Percent cover of embedded patches as a function of time for the three algae. The box plot horizontal bars show the median value, the box indicates the first and third QRs, and the whiskers indicate 1.5*IQR.
**Figure S3:** (a) Settlement of *Acropora cytherea* larvae on the subcryptic side of the PVC rings in the different algal treatments. (b) Survival of *Acropora cytherea* recruits initially settled on or in contact with live transplanted algae as a function of time for the different algal treatments. The control treatment is not shown due to the absence of live transplanted algae. The box plot horizontal bars show the median value, the box indicates the first and third QRs, and the whiskers indicate 1.5*IQR.
**Figure S4:** Rarefaction curves showing the number of observed ASVs as a function of sequencing depth for each sample. One *Lobophora* sample at T_0_ was excluded to low sequencing depth.
**Figure S5:** Relative abundance of ASVs at the family level in seawater samples from T_0_ to T_6_ and in algal samples in the different treatments at T_0_ and T_6_. Bars show the average relative abundance of all replicates. Numbers of replicates are shown in parenthesis on top of each bar.
**Figure S6:** Relative abundance of ASVs at the family level in coral larvae (T_0_) and coral recruits as a function of algal treatment and time (T_1_ to T_6_). Bars show the average relative abundance of all replicates. Numbers of replicates are shown in parenthesis on top of each bar.
**Figure S7:** NMDS ordination plot of the functional prediction of coral bacterial communities as a function of time.
**Figure S8:** Indicator metabolic pathways characterising coral larvae and coral recruits exposed to the different algal treatments as a function of time. Ctrl, control condition; Li, *Lithophyllum* sp; Lob, *Lobophora* spp. macroalgae; Nm, *N*. cf. *megalocystum*.
**Figure S9:** Symbiodiniaceae diversity and community composition of *Acropora cytherea* recruit samples as a function of algal treatment and time. (a) Alpha diversity (Shannon Index). The box plot horizontal bars show the median value, the box indicates the first and third QRs, and the whiskers indicate 1.5*IQR. (b) PCoA ordination based on Bray‐Curtis dissimilarities. (c) Relative abundance of ITS2 sequence types at the division level.
**Table S1:** Results of Kruskal‐Wallis tests on the effect of time on patch cover of the three tested algae. Post hoc tests are according to the Dunn's test adjusted with the Holm method. Significant *p*‐values (< 0.05) are in bold.
**Table S2:** Results of Kruskal‐Wallis tests on the effect of algal treatment on (a) the survival and (b) growth of *Acropora cytherea* recruits for the different time points. Post hoc comparisons are according to the Dunn's test adjusted with the Holm method. Significant *p*‐values (< 0.05) are in bold.
**Table S3:** Results of statistical models testing for the effect of sample type on the (a) alpha and (b) composition of bacterial communities. A Kruskal‐Wallis test was performed for the alpha diversity and a PERMANOVA based on Bray‐Curtis dissimilarities index was performed for the community composition. Post hoc comparisons are according to Dunn test for the Kruskal‐Wallis test and are adjusted with Benjamini & Hochberg for the PERMANOVA. Significant *p* values (< 0.05) are in bold.
**Table S4:** Results of statistical models testing for the effect of algal treatment and sampling time on the (a) alpha and (b) composition of the algal microbiome. A 2‐way parametric ANOVA was performed for the alpha diversity and a PERMANOVA based on Bray‐Curtis dissimilarities index was performed for the composition. Post hoc comparisons are according to Tukey's test for the parametric ANOVA and adjusted with Benjamini & Hochberg for the PERMANOVA. Significant *p* values (< 0.05) are in bold.
**Table S5:** Results of statistical models testing for the effect of algal treatment and sampling time on the (a) alpha and (b) composition of the recruit microbiome of *Acropora cytherea* . A 2‐way parametric ANOVA was performed for the alpha diversity and a PERMANOVA based on Bray‐Curtis dissimilarities index was performed for the composition. Alpha diversity data were rank transformed prior the analysis to meet the assumptions of the parametric ANOVA. Post hoc comparisons are adjusted with Benjamini & Hochberg for the PERMANOVA. Significant *p* values (< 0.05) are in bold.
**Table S6:** Result of PERMANOVA testing for the effect of time on the functional traits of *Acropora cytherea* bacterial communities. Post hoc comparisons were adjusted with Benjamini & Hochberg. Significant *p* values (< 0.05) are in bold.
**Table S7:** Results of statistical models testing for the effect of algal treatment and sampling time on the (a) alpha and (b) composition of Symbiodiniaceae of *A. cytherea* coral recruits. A 2‐way parametric ANOVA was performed for the alpha diversity and a PERMANOVA based on Bray‐Curtis dissimilarities index was performed for the composition.

## Data Availability

The data that support the findings of this study are openly available in BioProject at https://www.ncbi.nlm.nih.gov/bioproject/, reference number PRJNA1245259.
