# Supplementary material for: Impacts of Nearby Algae on Recruitment Success and Early Microbiome Development of the Coral Acropora cytherea
Source: Environ Microbiol. 2026 Jan 15;28(1):e70241. doi: 10.1111/1462-2920.70241 (PMC12807792; doi:10.1111/1462-2920.70241)
Supplement: Supplementary file 2 — Figure S1: Field photograph of PVC ring (80 mm diameter × 10 mm height) with embedded patches. PVC rings with algae were facing the reef substratum. Figure S2: Benthic community composition and algal patches cover on the inner surface of the PVC rings. (a) Benthic community composition at T0 and T6 for the different algal treatments. Thin and thick turf algae were defined as filamentous algae with heights of < 5 mm and ≥ 5 mm, respectively. Macroalgae were defined as non‐filamentous, anatomically complex algae. CCA = Crustose coralline algae. Values were assessed from 25 randomly chosen points on each ring using the PhotoQuad software. (b) Percent cover of embedded patches as a function of time for the three algae. The box plot horizontal bars show the median value, the box indicates the first and third QRs, and the whiskers indicate 1.5*IQR. Figure S3: (a) Settlement of Acropora cytherea larvae on the subcryptic side of the PVC rings in the different algal treatments. (b) Survival of Acropora cytherea recruits initially settled on or in contact with live transplanted algae as a function of time for the different algal treatments. The control treatment is not shown due to the absence of live transplanted algae. The box plot horizontal bars show the median value, the box indicates the first and third QRs, and the whiskers indicate 1.5*IQR. Figure S4: Rarefaction curves showing the number of observed ASVs as a function of sequencing depth for each sample. One Lobophora sample at T0 was excluded to low sequencing depth. Figure S5: Relative abundance of ASVs at the family level in seawater samples from T0 to T6 and in algal samples in the different treatments at T0 and T6. Bars show the average relative abundance of all replicates. Numbers of replicates are shown in parenthesis on top of each bar. Figure S6: Relative abundance of ASVs at the family level in coral larvae (T0) and coral recruits as a function of algal treatment and time (T1 to T6). Bars show the average r [file EMI-28-e70241-s001.docx]

**Supporting Information for**

**Impacts of nearby algae on recruitment success and early microbiome development of the coral *Acropora cytherea***

Camille Vizon^a,b^, Corentin Hochart^c^, Pierre E. Galand^c^, Maggy M. Nugues^a,d^

^a^ CRIOBE UAR 3278, EPHE-UPVD-CNRS-PSL, 52 Avenue Paul Alduy, 66860 Perpignan Cedex, France.

^b^ Institute for Chemistry and Biology of the Marine Environment (ICBM), Carl von Ossietzky Universität Oldenburg, 26129 Oldenburg, Germany

^c^ Sorbonne Université, CNRS, Laboratoire d’Ecogéochimie des Environnements Benthiques (LECOB), Observatoire Océanologique de Banyuls, 66650 Banyuls sur Mer, France.

^d^ Laboratoire d’Excellence « CORAIL», 98729 Papetoai, Moorea, French Polynesia.

**Corresponding authors:**

Camille Vizon


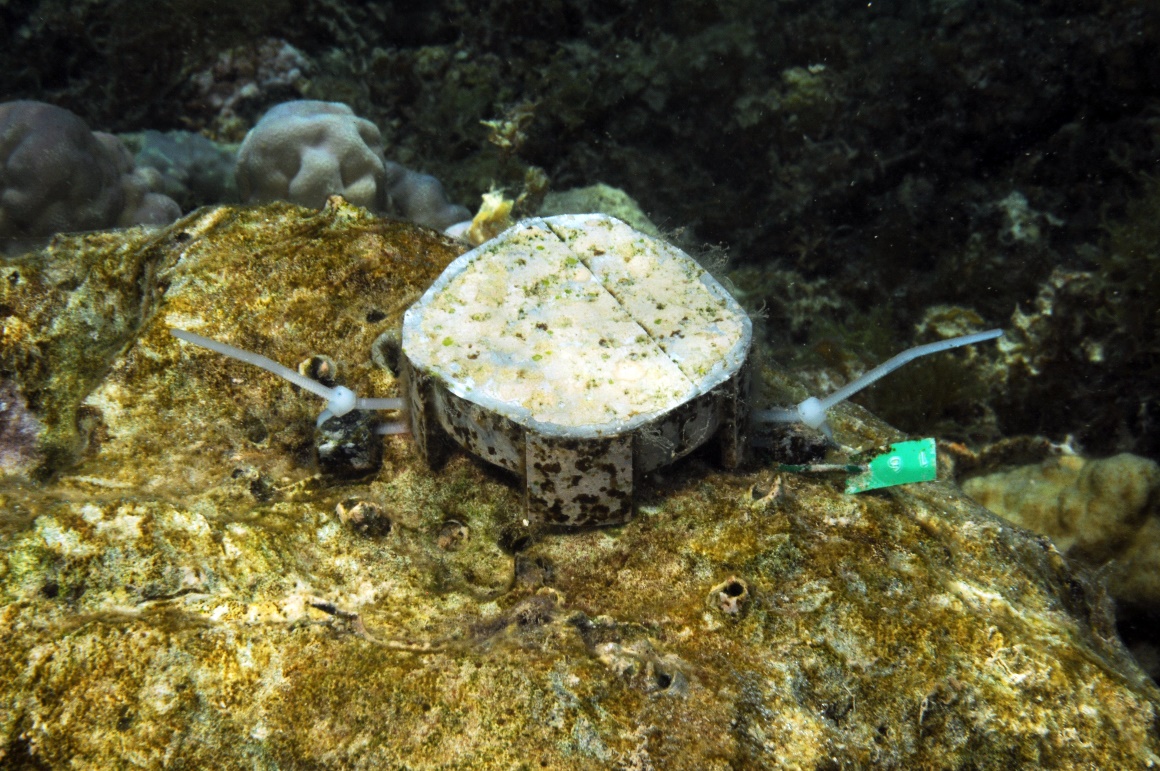
Figure S1: Field photograph of PVC ring (80 mm diameter x 10 mm height) with embedded patches. PVC rings with algae were facing the reef substratum.


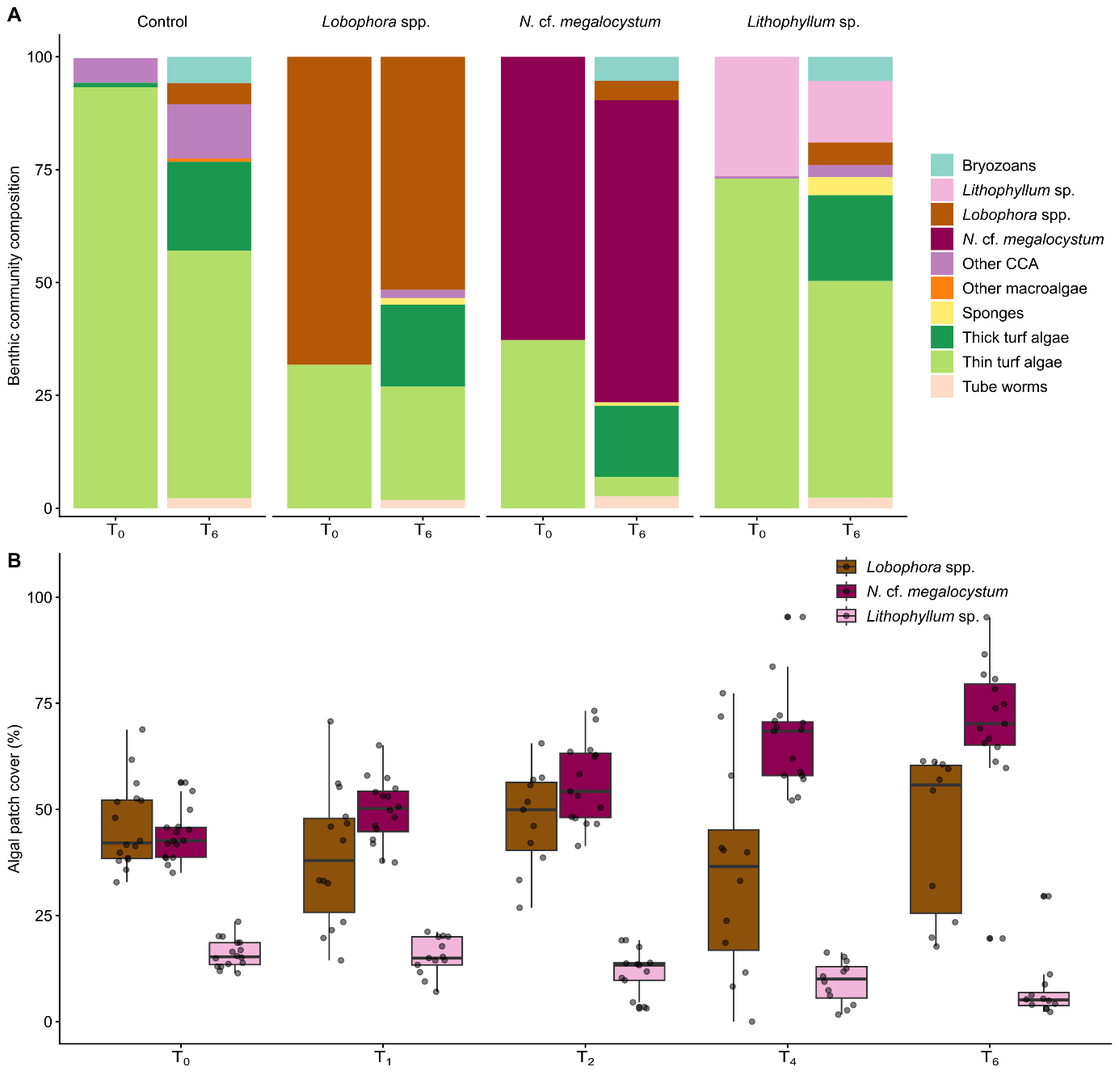
Figure S2: Benthic community composition and algal patches cover on the inner surface of the PVC rings. **(a)** Benthic community composition at T_0_ and T_6_ for the different algal treatments. Thin and thick turf algae were defined as filamentous algae with heights of < 5 mm and ≥ 5 mm, respectively. Macroalgae were defined as non-filamentous, anatomically complex algae. CCA = Crustose coralline algae. Values were assessed from 25 randomly chosen points on each ring using the PhotoQuad software. **(b)** Percent cover of embedded patches as a function of time for the three algae. The box plot horizontal bars show the median value, the box indicates the first and third QRs, and the whiskers indicate 1.5*IQR.


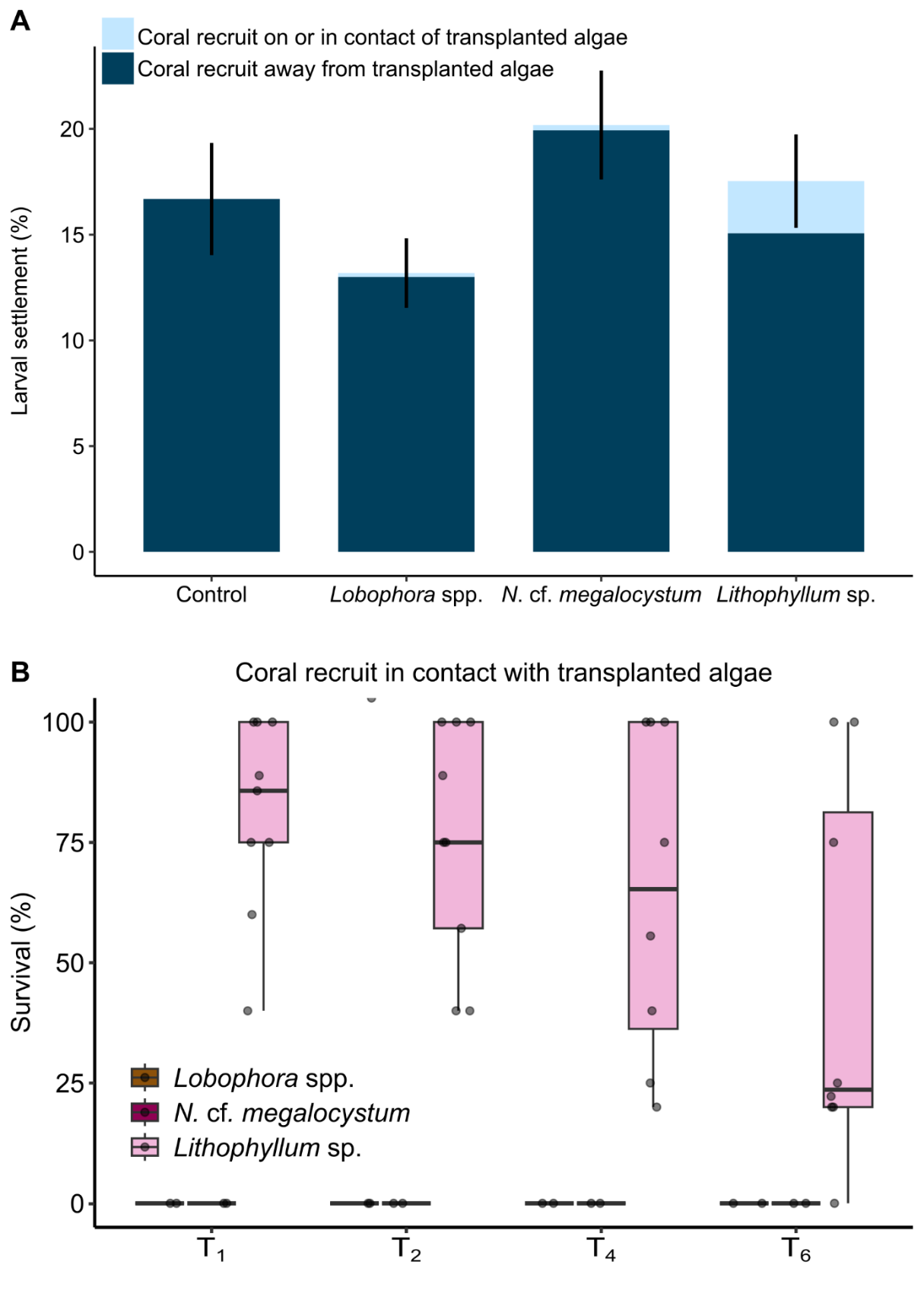


Figure S3: **(a)** Settlement of *Acropora cytherea* larvae on the subcryptic side of the PVC rings in the different algal treatments. **(b)** Survival of *Acropora cytherea* recruits initially settled on or in contact with live transplanted algae as a function of time for the different algal treatments. The control treatment is not shown due to the absence of live transplanted algae. The box plot horizontal bars show the median value, the box indicates the first and third QRs, and the whiskers indicate 1.5*IQR.


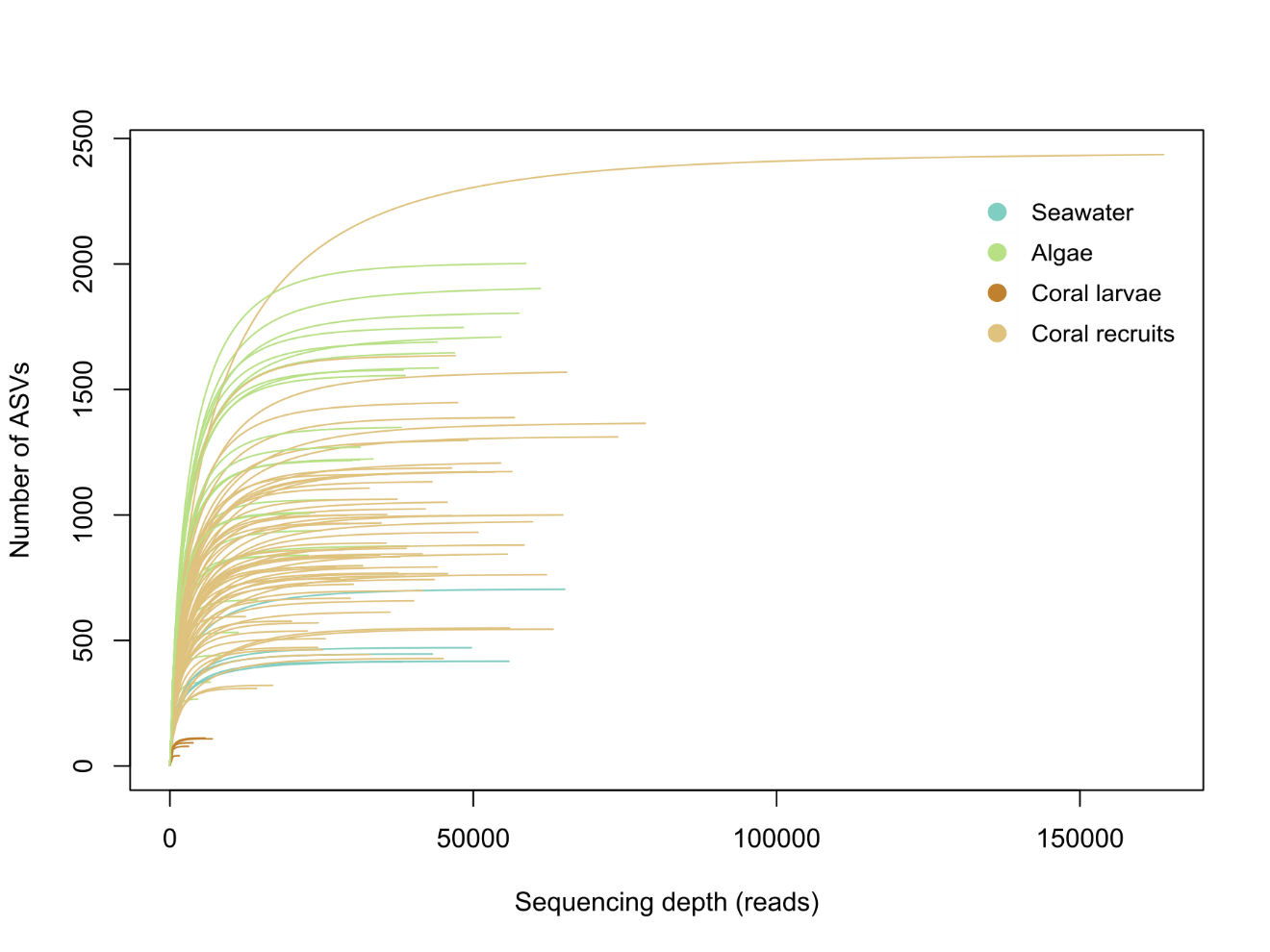


Figure S4: Rarefaction curves showing the number of observed ASVs as a function of sequencing depth for each sample. One *Lobophora* sample at T_0_ was excluded to low sequencing depth.


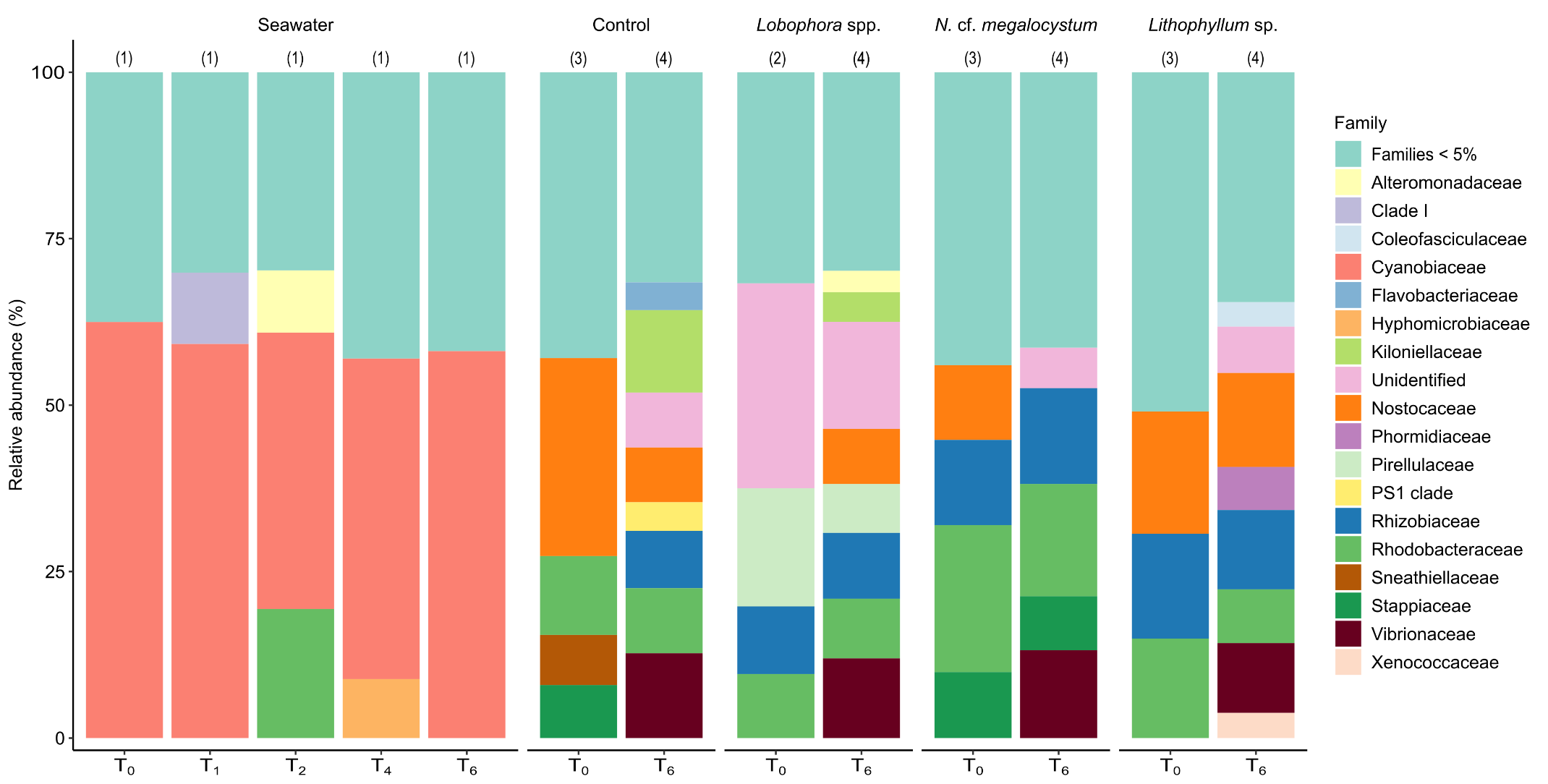


Figure S5: Relative abundance of ASVs at the family level in seawater samples from T_0_ to T_6_ and in algal samples in the different treatments at T_0_ and T_6_. Bars show the average relative abundance of all replicates. Numbers of replicates are shown in parenthesis on top of each bar.


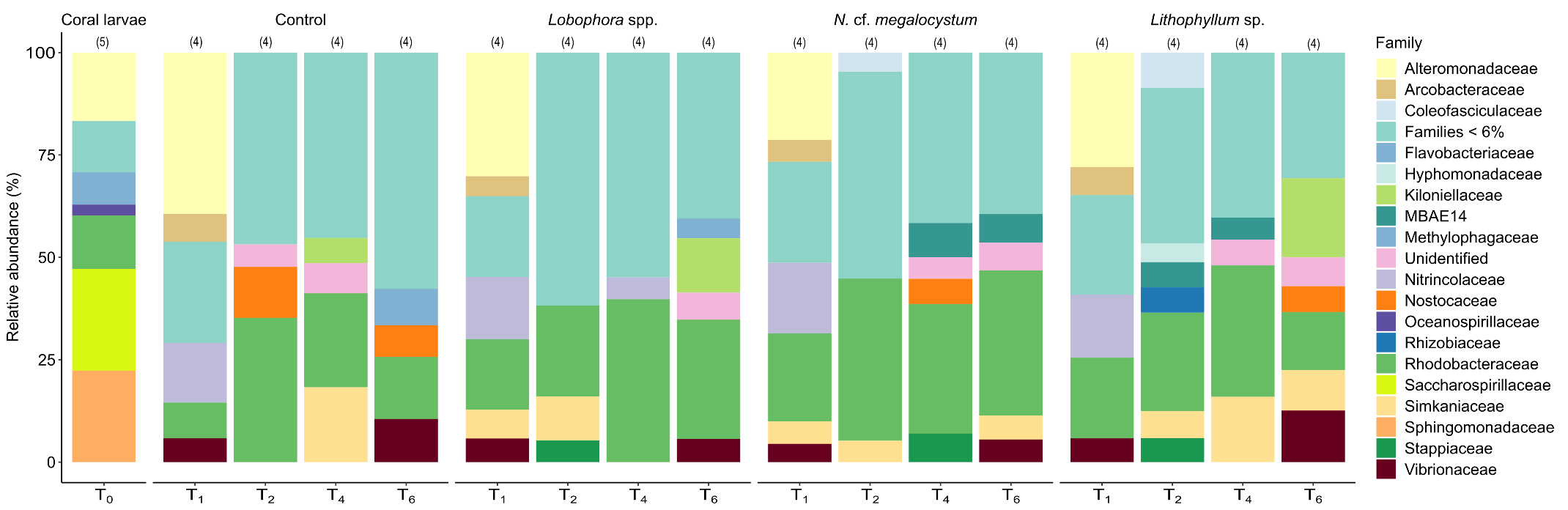
Figure S6: Relative abundance of ASVs at the family level in coral larvae (T_0_) and coral recruits as a function of algal treatment and time (T_1_ to T_6_). Bars show the average relative abundance of all replicates. Numbers of replicates are shown in parenthesis on top of each bar.


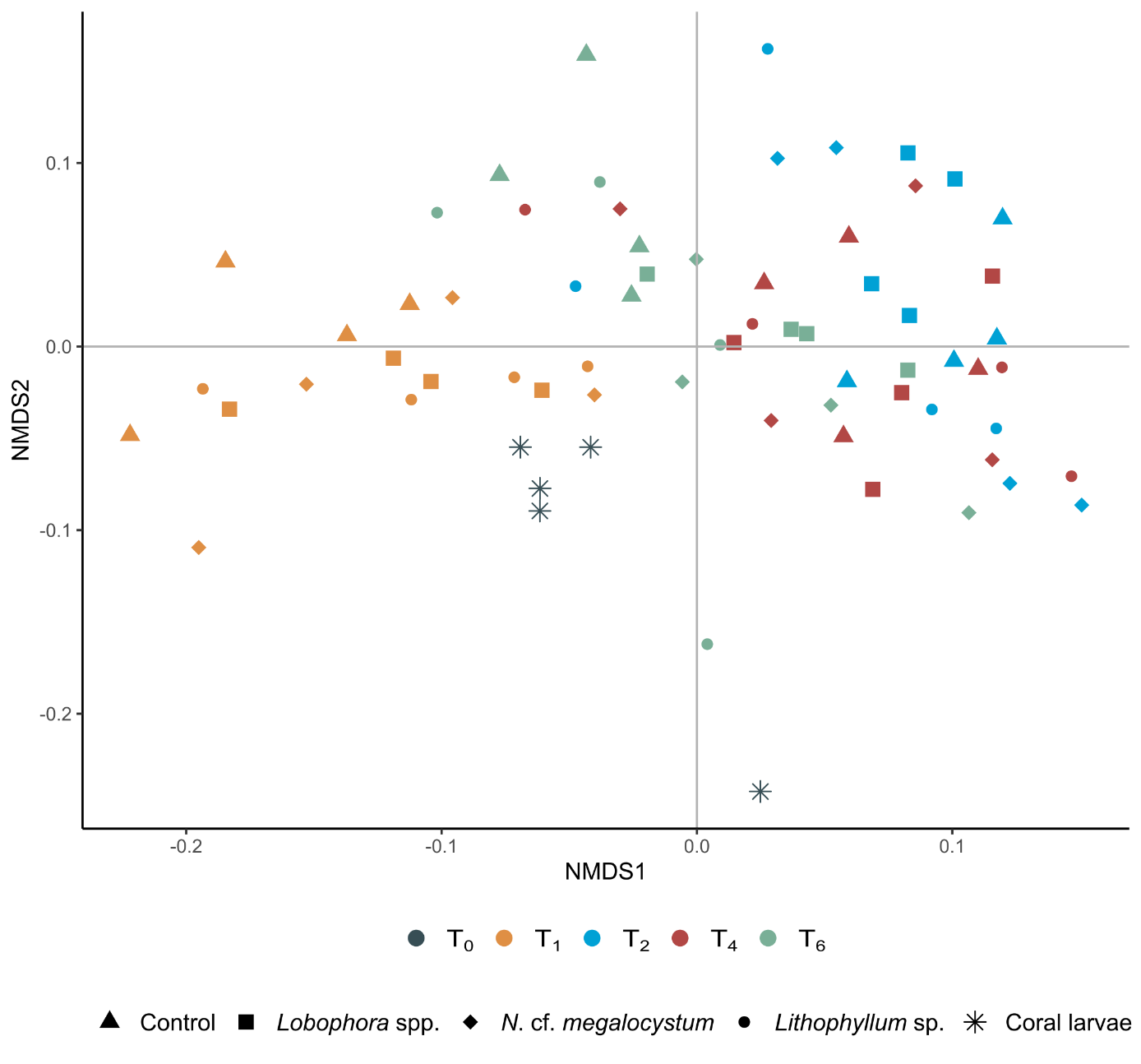
Figure S7**:** NMDS ordination plot of the functional prediction of coral bacterial communities as a function of time.


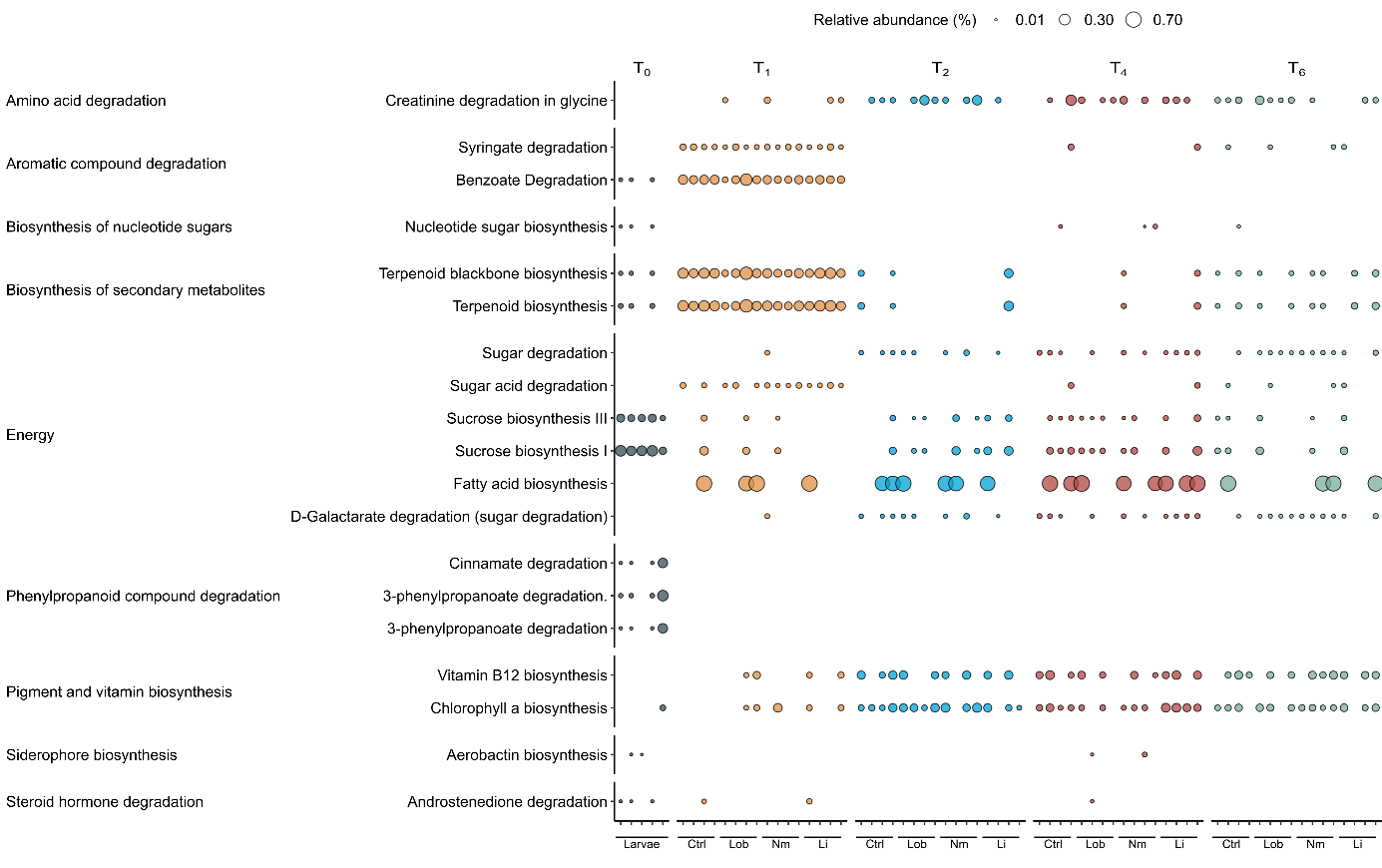
Figure S8: Indicator metabolic pathways characterizing coral larvae and coral recruits exposed to the different algal treatments as a function of time. Ctrl: control condition, Lob: *Lobophora* spp*.* macroalgae, Nm: *N.* cf. *megalocystum* and Li: *Lithophyllum* sp.


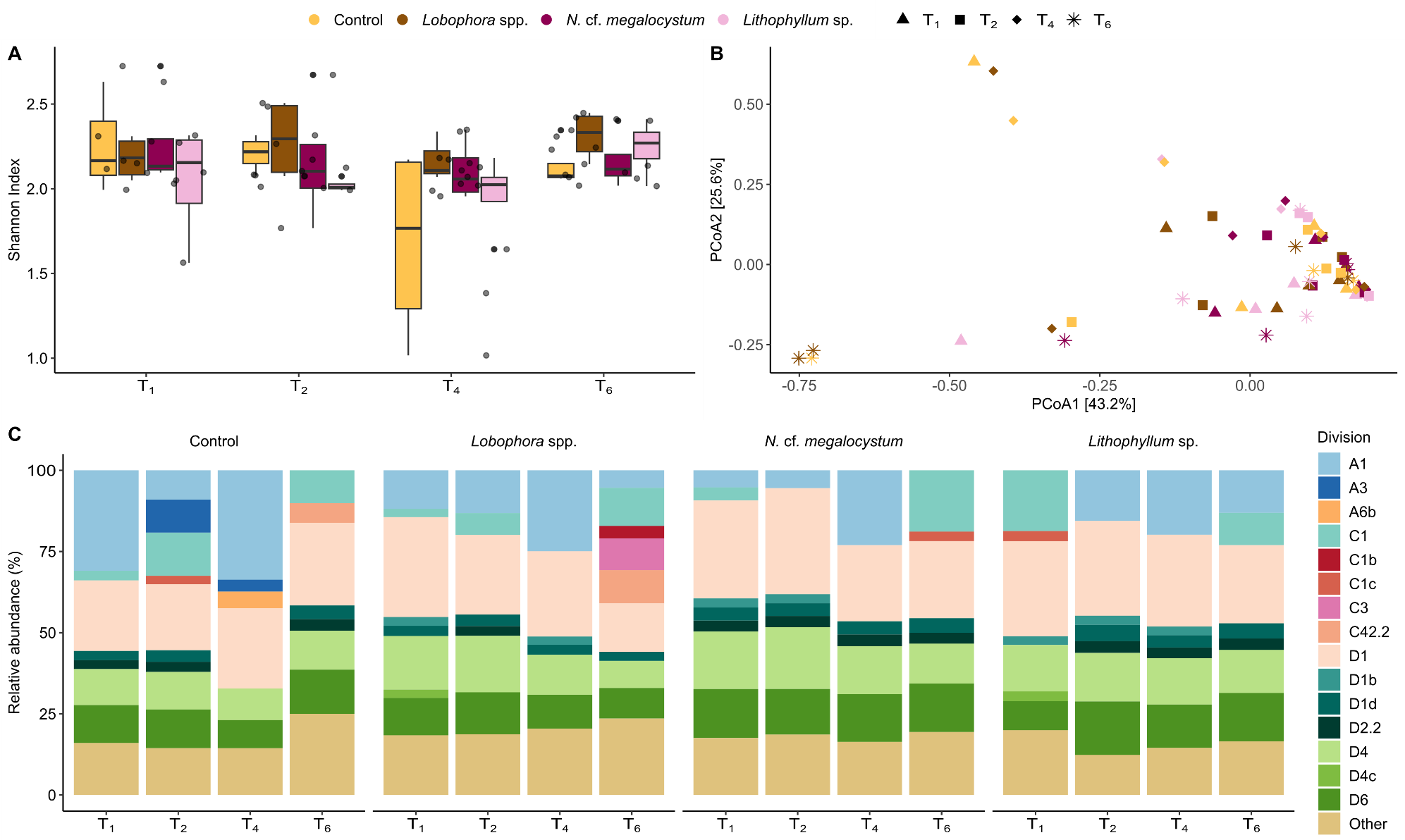
Figure S9: Symbiodiniaceae diversity and community composition of *Acropora cytherea* recruit samples as a function of algal treatment and time. **(a)** Alpha diversity (Shannon Index). The box plot horizontal bars show the median value, the box indicates the first and third QRs, and the whiskers indicate 1.5*IQR. **(b)** PCoA ordination based on Bray-Curtis dissimilarities. **(c)** Relative abundance of ITS2 sequence types at the division level.

Table S1: Results of Kruskal-Wallis tests on the effect of time on patch cover of the three tested algae. Post-hoc tests are according to the Dunn’s test adjusted with the Holm method. Significant p-values (< 0.05) are in bold.

| Source | df | F | p | Post-hoc comparisons | p |
| --- | --- | --- | --- | --- | --- |
| **a) *Lobophora* spp.** |  |  |  |  |  |
| Time | 4 | 4.38 | 0.357 |  |  |
| **b) *N.* cf. *megalocystum*** | | | | | |
| Time | 4 | 41.69 | **<0.001** | T_0_-T_1_ | 0.270 |
|  |  |  |  | T_0_-T_2_ | **0.016** |
|  |  |  |  | T_0_-T_4_ | **<0.001** |
|  |  |  |  | T_0_-T_6_ | **<0.001** |
|  |  |  |  | T_1_-T_2_ | 0.396 |
|  |  |  |  | T_1_-T_4_ | **0.006** |
|  |  |  |  | T_1_-T_6_ | **<0.001** |
|  |  |  |  | T_2_-T_4_ | 0.280 |
|  |  |  |  | T_2_-T_6_ | 0.067 |
|  |  |  |  | T_4_-T_6_ | 0.508 |
| **c) *Lithophyllum* sp.** | | | | | |
| Time | 4 | 23.79 | **<0.001** | T_0_-T_1_ | 0.808 |
|  |  |  |  | T_0_-T_2_ | 0.226 |
|  |  |  |  | T_0_-T_4_ | **0.019** |
|  |  |  |  | T_0_-T_6_ | **<0.001** |
|  |  |  |  | T_1_-T_2_ | 0.375 |
|  |  |  |  | T_1_-T_4_ | 0.054 |
|  |  |  |  | T_1_-T_6_ | **0.002** |
|  |  |  |  | T_2_-T_4_ | 0.920 |
|  |  |  |  | T_2_-T_6_ | 0.266 |
|  |  |  |  | T_4_-T_6_ | 0.666 |

Table S2: Results of Kruskal-Wallis tests on the effect of algal treatment on a) the survival and b) growth of *Acropora cytherea* recruits for the different time points. Post hoc comparisons are according to the Dunn’s test adjusted with the Holm method. Significant p-values (< 0.05) are in bold.

| Source | df | F | p | Post-hoc comparisons | p |
| --- | --- | --- | --- | --- | --- |
| **a) Survival** |  |  |  |  |  |
| T_1_ | 3 | 1.14 | 0.767 |  |  |
| T_2_ | 3 | 5.11 | 0.164 |  |  |
| T_4_ | 3 | 11.94 | **0.008** | Control - *Lobophora* spp*.* | 0.375 |
|  |  |  |  | Control - *N.* cf. *megalocystum* | 0.056 |
|  |  |  |  | Control – *Lithophyllum* sp. | **0.009** |
|  |  |  |  | *Lobophora* spp*.* - *N.* cf. *megalocystum* | 0.268 |
|  |  |  |  | *Lobophora* spp*.* - *Lithophyllum* sp. | 0.056 |
|  |  |  |  | *N.* cf. *megalocystum* - *Lithophyllum* sp. | 0.344 |
| T_6_ | 3 | 15.45 | **0.001** | Control - *Lobophora* spp*.* | 0.855 |
|  |  |  |  | Control - *N.* cf. *megalocystum* | **0.043** |
|  |  |  |  | Control - *Lithophyllum* sp. | **0.007** |
|  |  |  |  | *Lobophora* spp*.* - *N.* cf. *megalocystum* | **0.047** |
|  |  |  |  | *Lobophora* spp*.* - *Lithophyllum* sp. | **0.005** |
|  |  |  |  | *N.* cf. *megalocystum* - *Lithophyllum* sp. | 0.353 |
| **b) Growth** |  |  |  |  |  |
| T_1_ | 3 | 1.32 | 0.724 |  |  |
| T_2_ | 3 | 3.00 | 0.392 |  |  |
| T_4_ | 3 | 0.70 | 0.873 |  |  |
| T_6_ | 3 | 13.76 | **0.003** | Control - *Lobophora* spp*.* | 0.874 |
|  |  |  |  | Control - *N.* cf. *megalocystum* | 1.000 |
|  |  |  |  | Control - *Lithophyllum* sp. | **0.034** |
|  |  |  |  | *Lobophora* spp*.* - *N.* cf. *megalocystum* | 1.000 |
|  |  |  |  | *Lobophora* spp*.* - *Lithophyllum* sp. | **0.024** |
|  |  |  |  | *N.* cf. *megalocystum* - *Lithophyllum* sp. | **0.026** |
| **c) Survival (excluding coral recruit on or in contact with transplanted algae)** | | | | | |
| T_1_ | 3 | 0.47 | 0.925 |  |  |
| T_2_ | 3 | 4.07 | 0.254 |  |  |
| T_4_ | 3 | 8.10 | **0.044** | All pairwise comparisons | > 0.05 |
| T_6_ | 3 | 10.58 | **0.014** | Control - *Lobophora* spp*.* | 0.847 |
|  |  |  |  | Control - *N.* cf. *megalocystum* | **0.046** |
|  |  |  |  | Control - *Lithophyllum* sp. | 0.081 |
|  |  |  |  | *Lobophora* spp*.* - *N.* cf. *megalocystum* | 0.051 |
|  |  |  |  | *Lobophora* spp*.* - *Lithophyllum* sp. | 0.061 |
|  |  |  |  | *N.* cf. *megalocystum* - *Lithophyllum* sp. | 0.940 |
|  |  |  |  |  |  |

Table S3: Results of statistical models testing for the effect of sample type on the a) alpha and b) composition of bacterial communities. A Kruskal-Wallis test was performed for the alpha diversity and a PERMANOVA based on Bray-Curtis dissimilarities index was performed for the community composition. Post hoc comparisons are according to Dunn test for the Kruskal-Wallis test and are adjusted with Benjamini & Hochberg for the PERMANOVA. Significant p values (< 0.05) are in bold.

| Source | df | F | p | Post-hoc comparisons | p |
| --- | --- | --- | --- | --- | --- |
| **a) Alpha diversity (Shannon index)** | | | | |  |
| Sample type | 3 | 54.88 | **<0.001** | Algae vs Seawater | **<0.001** |
|  |  |  |  | Algae vs Coral larvae | **<0.001** |
|  |  |  |  | Algae vs Coral recruits | **<0.001** |
|  |  |  |  | Seawater vs Coral larvae | 0.538 |
|  |  |  |  | Seawater vs Coral recruits | **0.039** |
|  |  |  |  | Coral larvae vs Coral recruits | **0.008** |
| **b) Community composition based on Bray-Curtis dissimilarities** | | | | | |
| Sample type | 3 | 8.61 | **0.001** | Algae vs Seawater | **0.001** |
|  |  |  |  | Algae vs Coral larvae | **0.001** |
|  |  |  |  | Algae vs Coral recruits | **0.001** |
|  |  |  |  | Seawater vs Coral larvae | **0.012** |
|  |  |  |  | Seawater vs Coral recruits | **0.001** |
|  |  |  |  | Coral larvae vs Coral recruits | **0.001** |
| Residuals | 89 |  |  |  |  |

Table S4: Results of statistical models testing for the effect of algal treatment and sampling time on the a) alpha and b) composition of the algal microbiome. A 2-way parametric ANOVA was performed for the alpha diversity and a PERMANOVA based on Bray-Curtis dissimilarities index was performed for the composition. Post hoc comparisons are according to Tukey’s test for the parametric ANOVA and adjusted with Benjamini & Hochberg for the PERMANOVA. Significant p values (< 0.05) are in bold.

| Source | df | F | p | Post-hoc comparisons | | p |
| --- | --- | --- | --- | --- | --- | --- |
| **a) Alpha diversity (Shannon index)** | | | | |  |  |
| Algae | 3 | 14.54 | **<0.001** | Control - *Lobophora* spp*.*  Control – *N.* cf. *megalocystum*  Control – *Lithophyllum* sp.  *Lobophora* spp. - *N.* cf. *megalocystum*  *Lobophora* spp. - *Lithophyllum* sp.  *N.* cf. *megalocystum* - *Lithophyllum* sp. | | **<0.001** |
|  |  |  |  |  |  | 0.410 |
|  |  |  |  |  |  | 0.570 |
|  |  |  |  |  |  | **<0.001** |
|  |  |  |  |  |  | **0.009** |
|  |  |  |  |  |  | **0.043** |
| Time | 1 | 6.63 | **0.019** |  |  |  |
| Algae x Time | 3 | 1.57 | 0.230 |  |  |  |
| Residuals | 19 |  |  |  |  |  |
| **b) Composition based on Bray-Curtis dissimilarities** | | | | | | |
| Algae | 3 | 7.43 | **0.001** | Control - *Lobophora* spp*.*  Control - *N.* cf. *megalocystum*  Control - *Lithophyllum* sp.  *Lobophora* spp*.* - *N.* cf. *megalocystum*  *Lobophora* spp*.* - *Lithophyllum* sp.  *N.* cf. *megalocystum* - *Lithophyllum* sp. | | **0.024** |
|  |  |  |  |  |  | **0.024** |
|  |  |  |  |  |  | 0.600 |
|  |  |  |  |  |  | **0.012** |
|  |  |  |  |  |  | **0.006** |
|  |  |  |  |  |  | **0.040** |
| Time | 1 | 13.67 | **0.001** |  |  |  |
| Algae x Time | 3 | 2.55 | **0.001** | Control | T_0_ – T_6_ | **0.037** |
|  |  |  |  | *Lobophora* spp. | T_0_ – T_6_ | 0.067 |
|  |  |  |  | *N.* cf. *megalocystum* | T_0_ – T_6_ | **0.029** |
|  |  |  |  | *Lithophyllum* sp. | T_0_ – T_6_ | **0.033** |
|  |  |  |  | T_0_ | All pairwise comparisons | > 0.05 |
|  |  |  |  | T_6_ | All pairwise comparisons | **0.032** |
| Residuals | 19 |  |  |  |  |  |

Table S5: Results of statistical models testing for the effect of algal treatment and sampling time on the a) alpha and b) composition of the recruit microbiome of *Acropora cytherea*. A 2-way parametric ANOVA was performed for the alpha diversity and a PERMANOVA based on Bray-Curtis dissimilarities index was performed for the composition. Alpha diversity data were rank transformed prior the analysis to meet the assumptions of the parametric ANOVA. Post hoc comparisons are adjusted with Benjamini & Hochberg for the PERMANOVA. Significant p values (< 0.05) are in bold.

| Source | df | F | p | Post-hoc comparisons | p |
| --- | --- | --- | --- | --- | --- |
| **a) Alpha diversity (Shannon index)** | | | | |  |
| Algae | 3 | 0.72 | 0.546 |  |  |
| Time | 3 | 0.88 | 0.460 |  |  |
| Algae x Time | 9 | 1.40 | 0.214 |  |  |
| Residuals | 48 |  |  |  |  |
| **b) Composition based on Bray-Curtis dissimilarities** | | | | | |
| Algae | 3 | 1.15 | 0.104 |  |  |
| Time | 3 | 4.62 | **0.001** | T_1_ – T_2_ | **0.001** |
|  |  |  |  | T_1_ – T_4_ | **0.001** |
|  |  |  |  | T_1_ – T_6_ | **0.001** |
|  |  |  |  | T_2_ – T_4_ | **0.001** |
|  |  |  |  | T_2_ – T_6_ | **0.001** |
|  |  |  |  | T_4_ – T_6_ | **0.001** |
| Algae x Time | 9 | 1.03 | 0.298 |  |  |
| Residuals | 48 |  |  |  |  |

Table S6: Result of PERMANOVA testing for the effect of time on the functional traits of *Acropora cytherea* bacterial communities. Post hoc comparisons were adjusted with Benjamini & Hochberg. Significant p values (< 0.05) are in bold.

| Source | df | F | p | Post-hoc comparisons | p |
| --- | --- | --- | --- | --- | --- |
| Time | 4 | 10.95 | **0.001** | T_0_ - T_1_ | **0.003** |
|  |  |  |  | T_0_ - T_2_ | **0.016** |
|  |  |  |  | T_0_ - T_4_ | **0.012** |
|  |  |  |  | T_0_ - T_6_ | **0.010** |
|  |  |  |  | T_1_ - T_2_ | **0.003** |
|  |  |  |  | T_1_ - T_4_ | **0.003** |
|  |  |  |  | T_1_ - T_6_ | **0.008** |
|  |  |  |  | T_2_ - T_4_ | 0.517 |
|  |  |  |  | T_2_ - T_6_ | 0.254 |
|  |  |  |  | T_4_ - T_6_ | 0.422 |
| Residuals | 64 |  |  |  |  |

Table S7: Results of statistical models testing for the effect of algal treatment and sampling time on the a) alpha and b) composition of Symbiodiniaceae of *A. cytherea* coral recruits. A 2-way parametric ANOVA was performed for the alpha diversity and a PERMANOVA based on Bray-Curtis dissimilarities index was performed for the composition.

| Source | df | F | p |
| --- | --- | --- | --- |
| **a) Alpha diversity (Shannon index)** | | | |
| Algae | 3 | 1.33 | 0.276 |
| Time | 3 | 1.74 | 0.172 |
| Algae x Time | 9 | 0.70 | 0.710 |
| Residuals | 19 |  |  |
| **b) Composition based on Bray-Curtis dissimilarities** | | | |
| Algae | 3 | 1.26 | 0.235 |
| Time | 3 | 1.88 | 0.051 |
| Algae x Time | 9 | 0.64 | 0.962 |
| Residuals | 48 |  |  |
